# Spc2 modulates substrate- and cleavage site-selection in the yeast signal peptidase complex

**DOI:** 10.1083/jcb.202211035

**Published:** 2024-11-20

**Authors:** Yeonji Chung, Chewon Yim, Gilberto P. Pereira, Sungjoon Son, Lisbeth R. Kjølbye, Lauren E. Mazurkiewicz, Amy M. Weeks, Friedrich Förster, Gunnar von Heijne, Paulo C.T. Souza, Hyun Kim

**Affiliations:** 1 School of Biological Sciences and Institute of Biodiversity, Seoul National University, Seoul, South Korea; 2 Laboratoire de Biologie et Modélisation de la Cellule, CNRS, UMR 5239, Inserm, U1293, Université Claude Bernard Lyon 1, Ecole Normale Supérieure de Lyon, Lyon, France; 3 Centre Blaise Pascal de Simulation et de Modélisation Numérique, Ecole Normale Supérieure de Lyon, Lyon, France; 4 Molecular Microbiology and Structural Biochemistry, CNRS UMR 5086 and Université Claude Bernard Lyon 1, Lyon, France; 5 Department of Biochemistry, University of Wisconsin-Madison, Madison, WI, USA; 6 Structural Biochemistry, Bijvoet Centre for Biomolecular Research, Utrecht University, Utrecht, Netherlands; 7 Department of Biochemistry and Biophysics, Stockholm University, Stockholm, Sweden; 8 Science for Life Laboratory Stockholm University, Solna, Sweden

## Abstract

Secretory proteins are critically dependent on the correct processing of their signal sequence by the signal peptidase complex (SPC). This step, which is essential for the proper folding and localization of proteins in eukaryotic cells, is still not fully understood. In eukaryotes, the SPC comprises four evolutionarily conserved membrane subunits (Spc1–3 and Sec11). Here, we investigated the role of Spc2, examining SPC cleavage efficiency on various models and natural signal sequences in yeast cells depleted of or with mutations in Spc2. Our data show that discrimination between substrates and identification of the cleavage site by SPC is compromised when Spc2 is absent or mutated. Molecular dynamics simulation of the yeast SPC AlphaFold2-Multimer model indicates that membrane thinning at the center of SPC is reduced without Spc2, suggesting a molecular explanation for the altered substrate recognition properties of SPC lacking Spc2. These results provide new insights into the molecular mechanisms by which SPC governs protein biogenesis.

## Teaser

The mechanism by which the signal peptidase complex (SPC) distinguishes signal peptides from signal-anchored sequences remains unclear. Chung et al. demonstrate that the Spc2 subunit modulates the properties of the SPC and its surrounding membrane environment, enhancing the complex’s ability to discriminate between these sequences.

## Introduction

Secretory and membrane proteins destined for the secretory pathway have N-terminal cleavable signal peptides (SPs) or uncleavable signal-anchor sequences (SAs) that guide them to the SecYEG-YidC translocon in the bacterial plasma membrane and different types of Sec61-containing translocons in the endoplasmic reticulum (ER) membrane in eukaryotes (Hegde and Keenan, 2024). Upon targeting, both types of signal sequences help initiate co- or posttranslational protein translocation across the membrane. While translocons recognize both SPs and SAs, the membrane-integral signal peptidase complex (SPC) recognizes and cleaves SPs but not SAs.

SPs have a conserved tripartite structure: an N-terminal region containing basic residues (*n*-region), a hydrophobic core region (*h*-region), and a more polar C-terminal region that defines the cleavage site (*c*-region) (von Heijne, 1990). The *c*-region contains small, neutral amino acids in positions −1 and −3 and lacks proline in position +1 relative to the cleavage site (Perlman and Halvorson, 1983; von Heijne, 1984). SAs also have a positively charged *n*-region and a hydrophobic *h*-region but are not cleaved by SPC, even if they contain potential cleavage site motifs (Nilsson et al., 1994; Yim et al., 2018). Generally, both the *n*- and *h*-regions are longer in SAs than in SPs (Martoglio and Dobberstein, 1998; von Heijne, 1990), but how the SPC distinguishes these two types of signal sequences is not fully understood.

The eukaryotic SPC is composed of four subunits: Spc1/SPCS1, Spc2/SPCS2, Spc3/SPCS3, and Sec11/SEC11 (yeast/mammals) (Liaci et al., 2021). Sec11, which exists as two paralogs of SEC11 (A and C) in higher eukaryotes, is the catalytic subunit and shares sequence similarity with the *E*.*coli* signal peptidase I (LepB) (Shelness and Blobel, 1990; VanValkenburgh et al., 1999). Spc3 also shares sequence similarity with a part of LepB (Fang et al., 1997). While LepB appears to function as a monomer, SEC11 forms a heterotetramer with the other three SPC subunits (Liaci et al., 2021). The cryo-EM structure of the human SPC shows that the luminal domain of SPCS3 stabilizes the catalytic domain of SEC11A/C, together forming the catalytic core of the SPC (Liaci et al., 2021).

Notable differences between prokaryotic and eukaryotic signal peptidases are that the cytoplasmic side of the eukaryotic SPC is covered and that additional transmembrane helices (TMs) are contributed by Spc1/SPCS1 and Spc2/SPCS2. While Spc1/SPCS1 and Spc2/SPCS2 are not essential for growth or for the catalytic function in yeast (Mullins et al., 1996), we previously observed that incorrect cleavage of TM segments in membrane proteins was increased in a yeast Spc1 deletion strain (Yim et al., 2021).

Spc2 interacts with the β subunit of the Sec61 translocon in yeast and mammals, mediating transient interactions between the SPC and the Sec61 translocon (Antonin et al., 2000; Kalies et al., 1998). However, SPs of secretory precursors are efficiently processed in the absence of Spc2 in vivo (Mullins et al., 1996). Hence, the role of Spc2 as a connector between the SPC and the translocon is not essential and its function in the SPC still awaits to be defined.

In the present study, we prepared a *S*. *cerevisiae* Spc2 deletion strain as well as strains expressing mutant Spc2 subunits and assessed cleavage efficiencies of diverse types of signal sequences (SPs, SAs, and designed sequences having features intermediate between SPs and SAs) in these strains by pulse-labeling experiments to capture the early stages of protein maturation in the ER. Our data show that recognition of the substrate and identification of the cleavage site by SPC is altered when Spc2 is absent or mutated. Coarse-grained molecular dynamics (CGMD) simulations of the membrane-embedded AlphaFold2-Multimer model of the yeast SPC with and without Spc2 show that the membrane region at the center of the SPC, where the SP is presumed to be located prior to cleavage, is thicker when Spc2 is absent from the complex. These results suggest that Spc2 modulates the properties of SPC and its immediate membrane environment, thereby enhancing SPC’s ability to discriminate between SPs and SAs.

## Results

### N-length dependent signal sequence cleavage in spc2Δ cells

**Figure 1.**
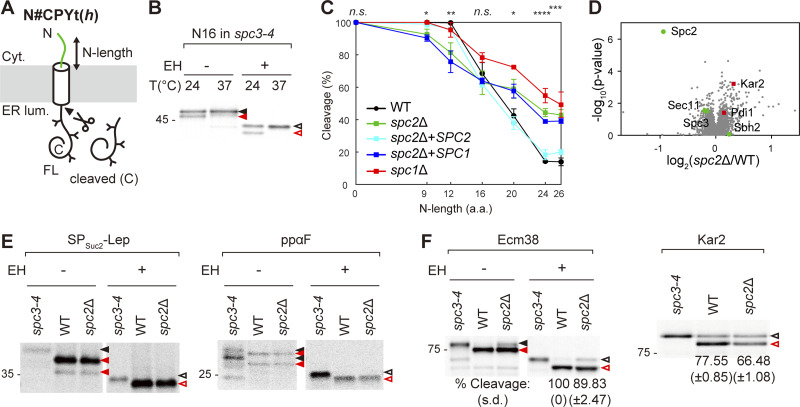
**The N-length-dependent signal sequence cleavage profile in the *spc2*Δ strain. (A)** Schematics of N#CPYt(*h*) constructs. The extended N-terminal sequences are from Dap2, a yeast SA protein (green). N# indicates the number of N-terminally extended residues, t indicates the C-terminal truncation after residue 323 of CPY, (*h*) denotes hydrophobic version of the CPY signal sequence (Table S1). N-glycan sites are indicated as Y. **(B)** N16CPYt(*h*) in the *spc3-4* strain was analyzed by pulse labeling at the indicated temperatures, subjected to endoglycosidase H treatment (EH) prior to SDS‒PAGE, and analyzed by autoradiography. **(C)** N#CPYt(*h*) constructs in the *spc2*Δ, *spc2*Δ + *SPC2*, and *spc2*Δ + *SPC1* strains were analyzed by pulse labeling. The relative amounts of cleaved products over total products (cleavage [%]) were plotted against the number of *n*-region residues (N-length). At least three independent experiments were carried out (*n* = 3/data point), and the average is shown with the standard deviation. P values between WT and *spc2*Δ and between *spc2*Δ and *spc2*Δ+*SPC2* strains were calculated by multiple two-tailed *t* tests; n.s., P > 0.05; *, P ≤ 0.05; **, P ≤ 0.01; ***, P ≤ 0.001; ****, P ≤ 0.0001. The cleavage profiles in the WT and *spc1*Δ strains (Yim et al., 2021) are shown in comparison. **(D)** The volcano plot of the WT and *spc2*Δ proteomes as quantified from mass spectrometry (Data S1). The relative abundances of Sec11, Spc3, Spc2, and Sbh2 are indicated in green circles and Pdi1 and Kar2 in red squares. **(E and F)** SP_Suc2_-Lep (Hessa et al., 2009) and ppαF, (F) Ecm38, Kar2 in the *spc3-4*, WT and *spc2*Δ strains were analyzed by pulse-labeling. Representative gels from at least three independent experiments are shown in F. Average cleavage efficiencies with standard deviation are indicated. Filled black and red arrows indicate glycosylated full-length and cleaved products, respectively, and unfilled black and red arrows indicate deglycosylated full-length and cleaved products, respectively. Source data are available for this figure: SourceData F1.

Previously, we prepared a set of carboxypeptidase Y (CPY) variants with signal sequences that differ in their *n*-region length and *h*-region hydrophobicity and established their cleavage profiles in *S*. *cerevisiae* (Yim et al., 2021). In the present study, we have analyzed a set of CPY variants with varying *n*-region length (N#CPYt(*h*)), Fig. 1 A and Table S1. We confirmed that the cleavage of N#CPYt(*h*) variants depends on the SPC by assessing the cleavage of N16CPYt(*h*) in the temperature-sensitive SPC catalytic mutant *spc3-4* strain (Fang et al., 1997). As expected, a SPC-cleaved, smaller-size band appeared at permissive (24°C) but not at non-permissive temperatures (37°C) (Fig. 1 B).

Signal sequences with N# ≤ 12 residues were fully processed during a 5-min pulse of [^35^S]-Met, whereas those with longer N-lengths were progressively less efficiently cleaved in the wild-type (WT) strain, indicating that the N-length influences cleavage efficiency (Fig. 1 C, black trace) (Yim et al., 2021). Interestingly, we observed a much weaker dependence on N-length when cleavage of N#CPYt(*h*) variants was assessed in cells lacking Spc2 (*spc2*Δ): shorter N-lengths (N# = 9, 12) were less efficiently cleaved, while longer N-lengths (N# = 20, 24, 26) were more efficiently cleaved, compared with those in WT cells (Fig. 1 C, green trace). Expression of Spc2 in the *spc2*Δ cells restored the cleavage efficiencies to the WT levels (Fig. 1 C, light blue trace).

We previously observed that N#CPYt(*h*) variants with longer N-lengths were more efficiently cleaved in the *spc1*Δ strain than in the WT strain, while—in contrast to the *spc2*Δ strain—shorter N-lengths were unaffected (Fig. 1 C, red trace) (Yim et al., 2021). This prompted us to check whether exogenous expression of Spc1 in *spc2*Δ cells could reduce the cleavage efficiencies of longer N#CPYt(*h*) variants to the WT level. However, overexpression of Spc1 did not complement the cleavage phenotype of the *spc2* deletion (Fig. 1 C, green trace), showing that Spc1 and Spc2 are not interchangeable. These data are in line with earlier findings that Spc1 and Spc2 have distinct functions (Mullins et al., 1996).

It has been reported that the amounts of the other subunits of SPC and Sbh2, the β subunit of the Ssh1 complex (a Sec61 complex homolog), were reduced in *spc2*Δ cells (Antonin et al., 2000). We therefore compared the levels of these proteins in the WT and *spc2*Δ cells by quantitative mass spectrometry (Fig. 1 D). The volcano plot shows that the abundance of Sec11 and Spc3 in the *spc2*Δ cells was reduced by ∼10% compared with WT cells, whereas no significant difference was observed for Sbh2. Spc1 was not found among the 2,676 proteins identified (Data S1), so we could not quantitate its abundance. Despite the mild reduction in the amount of the catalytic core subunits Sec11 and Spc3, the SPs of two secretory precursors, a Suc2 fusion protein (SP_Suc2_-Lep) and prepro α-factor (ppαF), were efficiently processed in both WT and *spc2*Δ cells, indicating that the catalytic activity of the SPC per se is at best marginally impaired in the absence of Spc2 (Fig. 1 E).

To validate the observations made with the engineered CPY variants, we tested the signal sequence cleavage of yeast proteins possessing relatively short *n*-region signal sequences (Ecm38 and Kar2, both have SPs with a 10 residues long *n*-region), Fig. 1 F. Consistent with the CPY results, cleavage of Ecm38 and Kar2 was decreased in the *spc2*Δ cells compared with WT cells. These data show that Spc2 acts to promote cleavage of signal sequences with short *n*-regions (N# < 16) and reduce cleavage of those with long *n*-regions (N# > 16), suggesting that Spc2 helps sharpen the discrimination between SPs and SAs.

### The C-terminal domain of Spc2 is important for N-length dependent signal sequence cleavage

**Figure 2.**
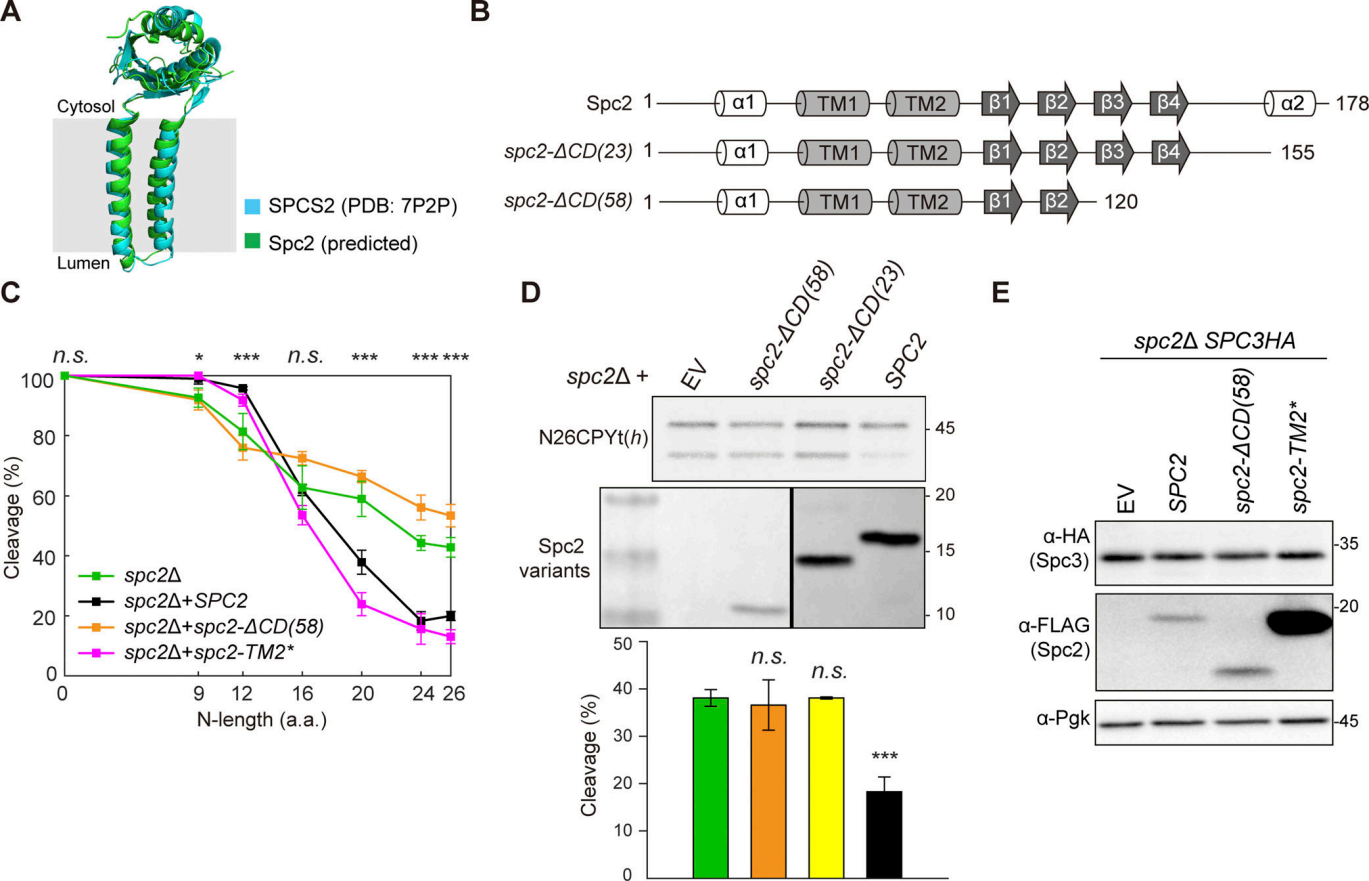
**The C-terminal domain of Spc2 is important for N-length-dependent signal sequence cleavage. (A)** Structures of human SPCS2 (PDB: 7P2P) and yeast Spc2 (predicted by AlphaFold2, UniProt ID Q04969) are overlaid. **(B)** Secondary structures of the predicted Spc2. **(C)** Cleavage efficiencies of N#CPYt(*h*) variants in *spc2*Δ cells with *SPC2*, *spc2-ΔCD*(*58*), and spc2-TM2*. Data of N#CPYt(*h*) variants in the *spc2*Δ cells in Fig. 1 (C) are shown for comparison. At least three independent experiments were carried out (*n* = 3/data point), and the average is shown with the standard deviation. P values between *spc2*Δ+*SPC2* and *spc2*Δ+ *spc2-ΔCD*(*58*) strains were calculated by multiple two-tailed *t* tests; n.s., P > 0.05; *, P ≤ 0.05; **, P ≤ 0.01; ***, P ≤ 0.001; ****, P ≤ 0.0001. **(D)** Cleavage efficiency of N26CPYt(*h*) in *spc2*Δ cells with EV, *spc2-ΔCD*(*58*), *spc2-ΔCD*(*23*) or *SPC2* (under the GPD promoter). The expression levels of Spc2 in the indicated strains were assessed by western blotting using anti-FLAG antibodies recognizing Spc2-FLAG. At least three independent experiments were carried out (*n* = 3/data point), and the average is shown with the standard deviation. *P* values were calculated by multiple two-tailed *t* tests; n.s., P > 0.05; ***, P ≤ 0.001. **(E)** Whole-cell lysates from the *spc2Δ*,*SPC3HA* strain carrying an empty vector (EV), *SPC2* (under its own promoter), *spc2-ΔCD*(*58*), and spc2-TM2* were analyzed by western blotting. PgK is a loading control. Source data are available for this figure: SourceData F2.

**Figure S1.**
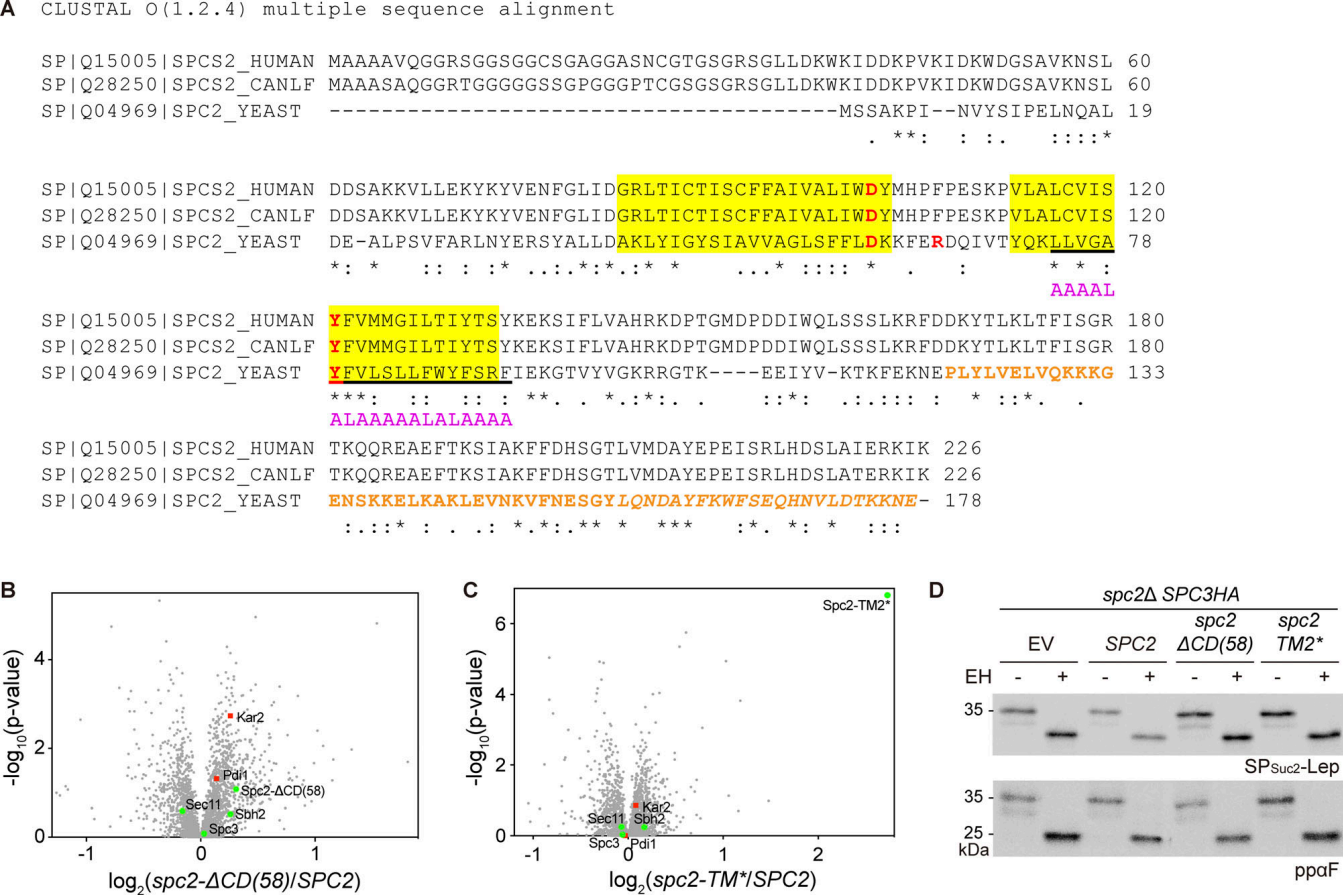
**Presents sequences and data related to Spc2, its homologs and mutants. (A)** Sequence alignment of Spc2 homologs (human, canine and *S*. *cerevisiae*). Predicted TMs are in yellow. Underlined sequences were replaced with the 4L/15A hydrophobic segment in Spc2-TM2* (shown in magenta). Orange colored sequences were truncated in Spc2-ΔCD(58) and orange colored sequences in italic were truncated in Spc2-ΔCD(23). **(B and C)** The volcano plots comparing the proteomes from *spc2*Δ cells carrying *SPC2* and *spc2-ΔCD*(*58*) (B) or *SPC2* and spc2-TM2* (C) as quantified from mass spectrometry (Data S2). The relative abundances of Sec11, Spc3, Spc2, and Sbh2 are indicated in green circles, and Pdi1 and Kar2 in red square. **(D)** Cleavage of SP_Suc2_-Lep (Hessa et al., 2009) and ppαF in the *spc2*Δ, *SPC3HA* strain carrying an empty vector (EV), *SPC2*, *spc2-ΔCD*(58), and spc2-TM2* was analyzed by pulse labeling. Source data are available for this figure: SourceData FS1.

The human SPCS2 and the Alphafold2-predicted yeast Spc2 structures are well conserved between yeast and human and constitute most of the cytosolic part of SPC (Fig. 2 A). Prior to cleavage by SPC, signal sequences are inserted in the ER membrane in the N_cytosol_-C_lumen_ orientation. Thus, the *n*-region faces the cytosol and likely encounters the cytosolic domain of Spc2. To test if the cytosolic C-terminal domain of Spc2 affects the N-length-dependent substrate selection by SPC, we constructed Spc2 mutants lacking the C-terminal 58 or 23 residues of Spc2 (Spc2-ΔCD(58), Spc2-ΔCD(23)), Fig. 2 B, along with another mutant having the intact C-terminal domain but with TM2 replaced by a 19-residue long transmembrane segment composed of Ala and Leu residues (Spc2-TM2*), Fig. S1.

The cleavage pattern of the N#CPYt(*h*) variants in the *spc2*Δ cells carrying spc2-TM2* was similar to that in cells carrying *SPC2* (Fig. 2 C). In contrast, in *spc2*Δ cells carrying *spc2-ΔCD*(*58*), shorter N-length variants were less efficiently cleaved, while longer N-length variants were more efficiently cleaved than in WT cells, similar to the cleavage profile of *spc2*Δ cells (Fig. 2 C). Increased cleavage efficiency was also seen for N26CPYt(*h*) in *spc2*Δ cells carrying *spc2-ΔCD*(*23*), a shorter truncation of the C-terminus (Fig. 2 D).

To check the stability of Spc3 in the Spc2 mutant cells, a sequence encoding three copies of a hemagglutinin (HA) tag was fused at the 3′ end of the *SPC3* gene in the *spc2*Δ strain (*spc2*Δ*SPC3HA*). *SPC2*, *spc2-ΔCD*(58), spc2-TM2*, and empty vector (EV) were transformed into the *spc2*Δ*SPC3HA* strain, and steady state levels of Spc3 and the Spc2 variants were assessed by western blotting (Fig. 2 E). Spc2-ΔCD(58) and Spc2-TM2* were expressed at the expected sizes and the expression level of endogenous Spc2 or higher. The expression levels of Spc3 in these cells were unaffected. The relative protein abundances of Sec11, Spc3, Spc2, and Sbh2 between the *spc2*Δ cells carrying *SPC2* and *spc2-ΔCD*(58) or between those cells carrying *SPC2* and spc2-TM2* were also assessed by quantitative mass spectrometry, and we found no statistically significant differences between them (Fig. S1, B and C; and Data S2). Further, the SPs of the two secretory proteins SP_Suc2_-Lep and ppαF were efficiently cleaved in the *spc2*Δ cells harboring either of the Spc2 mutants (Fig. S1 D). These data indicate that the stability of the SPC catalytic core subunits and their activity remain unaltered upon truncation of the Spc2 C-terminus or replacement of its TM2.

Notably, quantitative mass spectrometry data showed that the abundance of Kar2 and Pdi1, two ER chaperones, was markedly increased in the *spc2*Δ and *spc2-ΔCD*(58) strains, but not in the spc2-TM2* strain (Fig. 1 D; and Fig. S1, B and C). These data suggest that the ER unfolded protein response (UPR) is triggered when N-length-dependent substrate discrimination is compromised. We conclude that the C-terminal domain of Spc2 is a key determinant for the N-length-dependent discrimination between SPs and SAs.

### Effects of the Spc2 deletion on cleavage of TM segments

**Figure 3.**
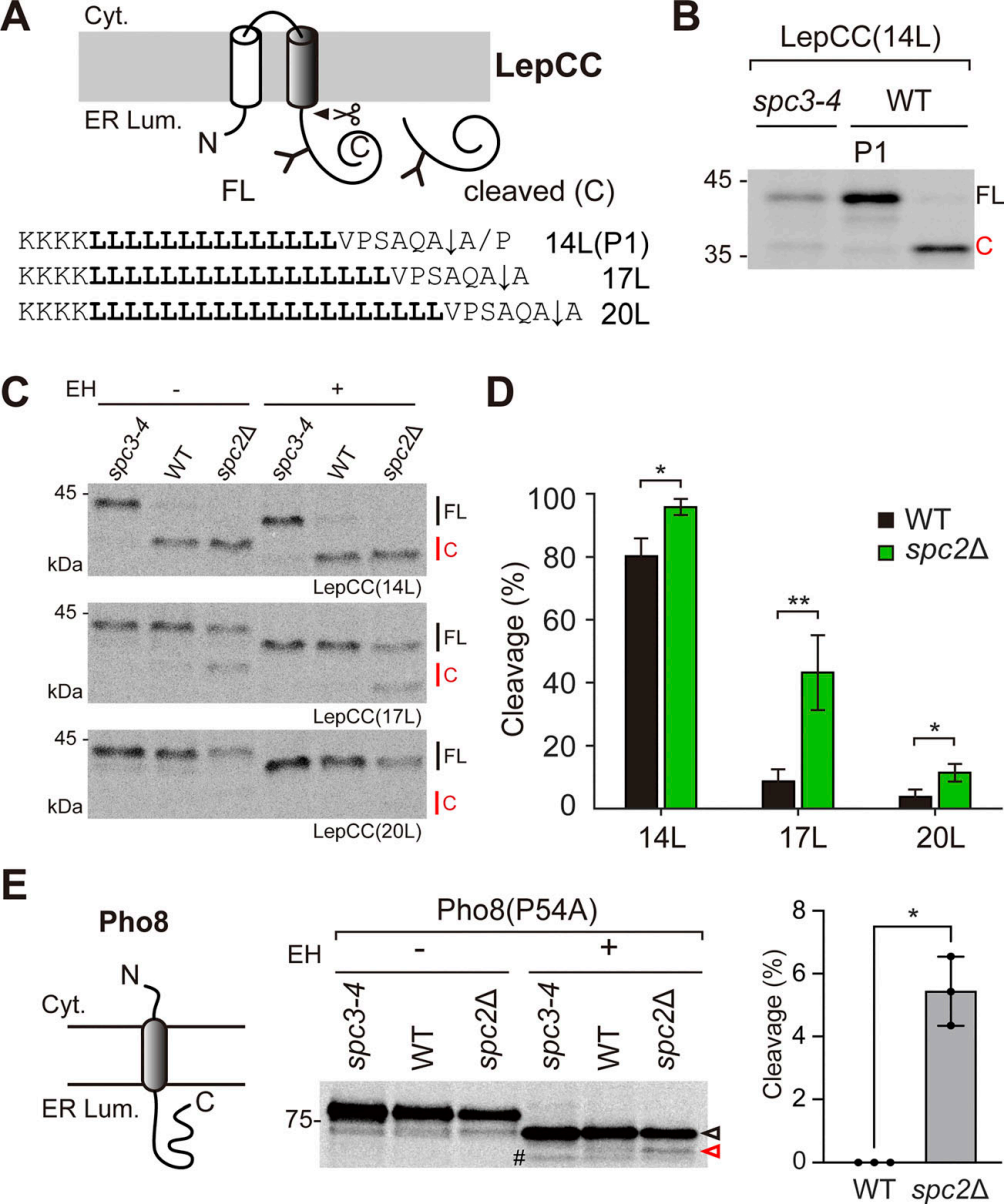
**Effects of Spc2 deletion on the cleavage of TM segments. (A)** Schematics and sequences of LepCC variants. The Leu TM segment is in bold, and the cleavage site is indicated as ↓. LepCC(14L(P1)) carries an A to P mutation in the +1 position of the cleavage site, and the cleavage is inhibited. **(B)** LepCC(14L) in the *spc3-4* and WT strains and LepCC(14L[P1]) in the WT strain were analyzed by 5 min pulse labeling, and Endo H (EH) was added to the samples prior to SDS‒PAGE. **(C)** The indicated LepCC variants in the *spc3-4*, WT, and *spc2*Δ strains were analyzed by pulse labeling. Protein samples were treated with or without Endo H (EH) prior to SDS‒PAGE. FL, full-length; C, cleaved species. **(D)** The relative amounts of cleaved products over total products (cleavage [%]) were plotted (*n* = 3). **(E)** Schematic of Pho8 and its SA sequence (in bold) plus 5 downstream residues. The mutated Pro54 residue is indicated in italics. A representative gel is shown. Average cleavage efficiencies from three independent experiments (*n* = 3/datapoint) and standard deviation are shown. Unfilled black and red arrows indicate de-glycosylated full-length and cleaved products, respectively. ^#^ indicates a nonspecific band. P values were calculated by two-tailed unpaired *t* test with Welch’s correction; P > 0.05; *. Source data are available for this figure: SourceData F3.

As shown above, N#CPYt(*h*) variants with N# > 16 were more efficiently cleaved when Spc2 was absent or lacking its C-terminal domain. This led us to ask whether Spc2 deletion or truncation also affects the cleavage of TM segments. To address this question, a set of model substrates based on an *E*. *coli* LepB construct called LepCC (Nilsson et al., 1994; Yim et al., 2021) was used. The LepCC variants possess an engineered TM segment with *h*-regions composed of 14, 17, or 20 Leu residues, followed by a signal sequence cleavage cassette (Fig. 3 A). Glycosylation of an N-linked glycosylation site in the C-terminal domain indicates proper translocation of the C-terminal domain across the ER membrane, and processing by SPC can be detected as the appearance of a smaller size band on SDS-gels (Nilsson et al., 1994). LepCC(14L) was pulse-labeled in the *spc3-4* and WT strains, along with the non-cleavable mutant LepCC(14L(P1)) (Fig. 3 B). Expression of LepCC(14L) in the *spc3-4* strain and LepCC(14L(P1)) in the WT strain resulted in the full-length product, while LepCC(14L) in the WT strain was efficiently processed, indicating that LepCC(14L) undergoes SPC-mediated cleavage.

The cleavage efficiencies of LepCC variants in the *spc3-4*, WT, and *spc2*Δ strains were assessed by 5-min pulse labeling (Fig. 3, C and D). In WT cells, LepCC(14L) was efficiently cleaved, while LepCC(17L) and LepCC(20L) were not. However, in *spc2*Δ cells, the cleavage efficiency of all LepCC variants was increased compared with that in WT cells, with the most pronounced difference observed for the LepCC(17L) variant (approximately threefold increase in cleavage efficiency).

**Figure S2.**
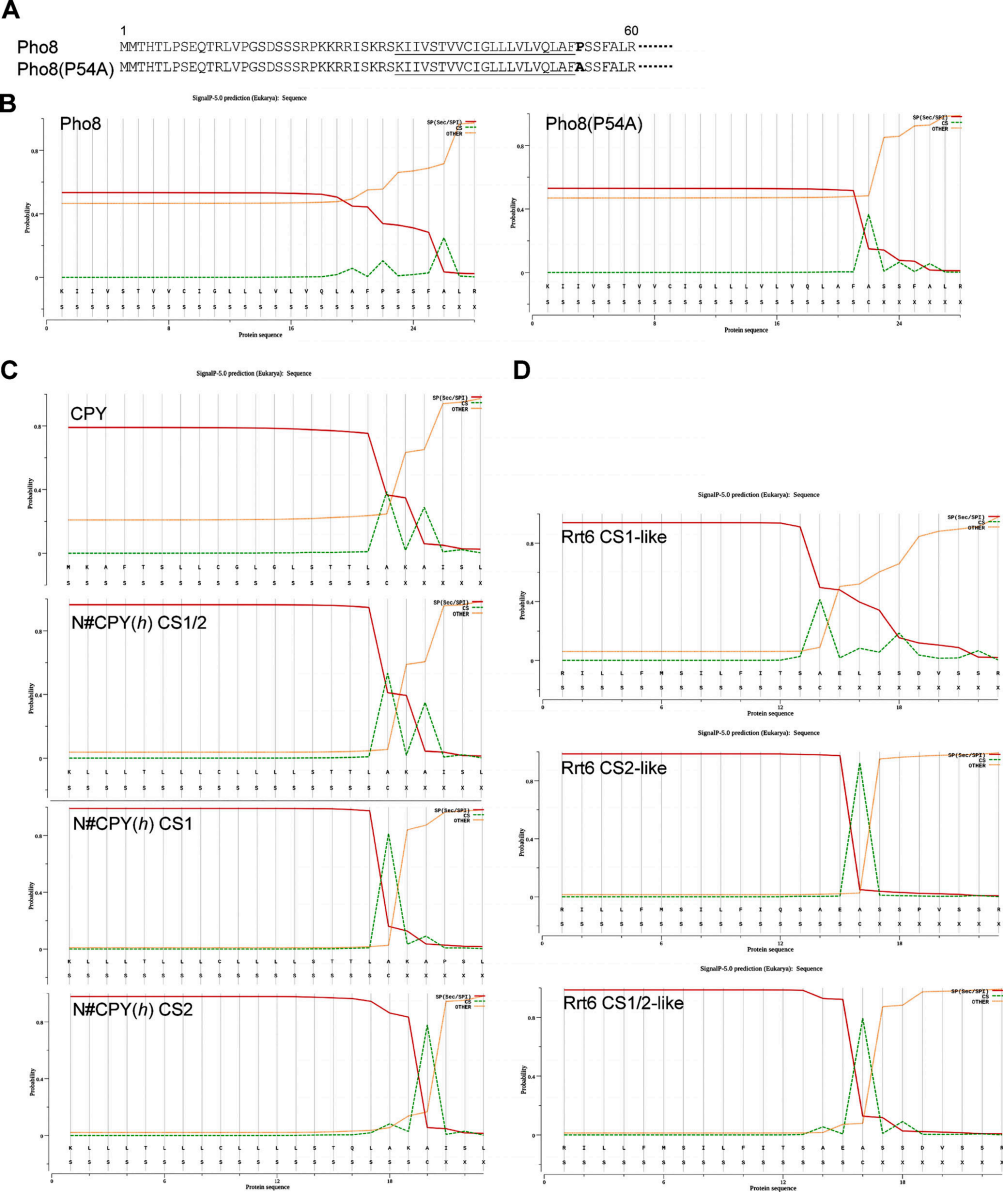
**Shows the predicted signal sequence cleavage sites of the proteins used in the study. (A)** The N-terminal 60 residues of Pho8 and Pho8(P54A) are shown. Signal-anchored sequences are underlined. **(B–D)** SignalP (Almagro Armenteros et al., 2019) predictions for Pho8 and Pho8(P54A) (B), CPY, N#CPYt(*h*) CS1/2, CS1 and CS2 (C). Rrt6 (CS1-like), Rrt6_T48Q,L52A,D55P (CS2-like), Rrt6_L52A (CS1/2-like) (D).

To verify these observations using a natural membrane protein, we assessed the SPC-mediated cleavage of the SA protein Pho8, a vacuolar alkaline phosphatase (Fig. 3 E). Pho8 contains a single N-terminal hydrophobic TM with a ∼20-amino acid-long *h*-region and weakly predicted potential cleavage sites at the C-terminal end (Fig. S2, A and B). We introduced a single point mutation (P54A) at the C-terminal end of the SA sequence to increase the predicted cleavage efficiency (Fig. S2). We observed a slight but consistent increase of the cleaved product in the *spc2*Δ strain but not in the WT strain upon 5-min pulse labeling (Fig. 3 E).

Thus, the deletion of Spc2 enhances the cleavage efficiencies of TM segments, particularly of those that have slightly longer *h*-regions than those in typical SPs (Petersen et al., 2011), again underscoring the importance of Spc2 for substrate discrimination by SPC.

### Effects of the Spc2 deletion on cleavage site selection

SPs often contain more than one potential SPC cleavage site in the *c*-region, and certain SPs are naturally cleaved at multiple sites (von Heijne, 1984). However, it is unknown how the preferred cleavage site is selected by SPC and whether Spc1 and Spc2 affect cleavage site selection.

**Figure 4.**
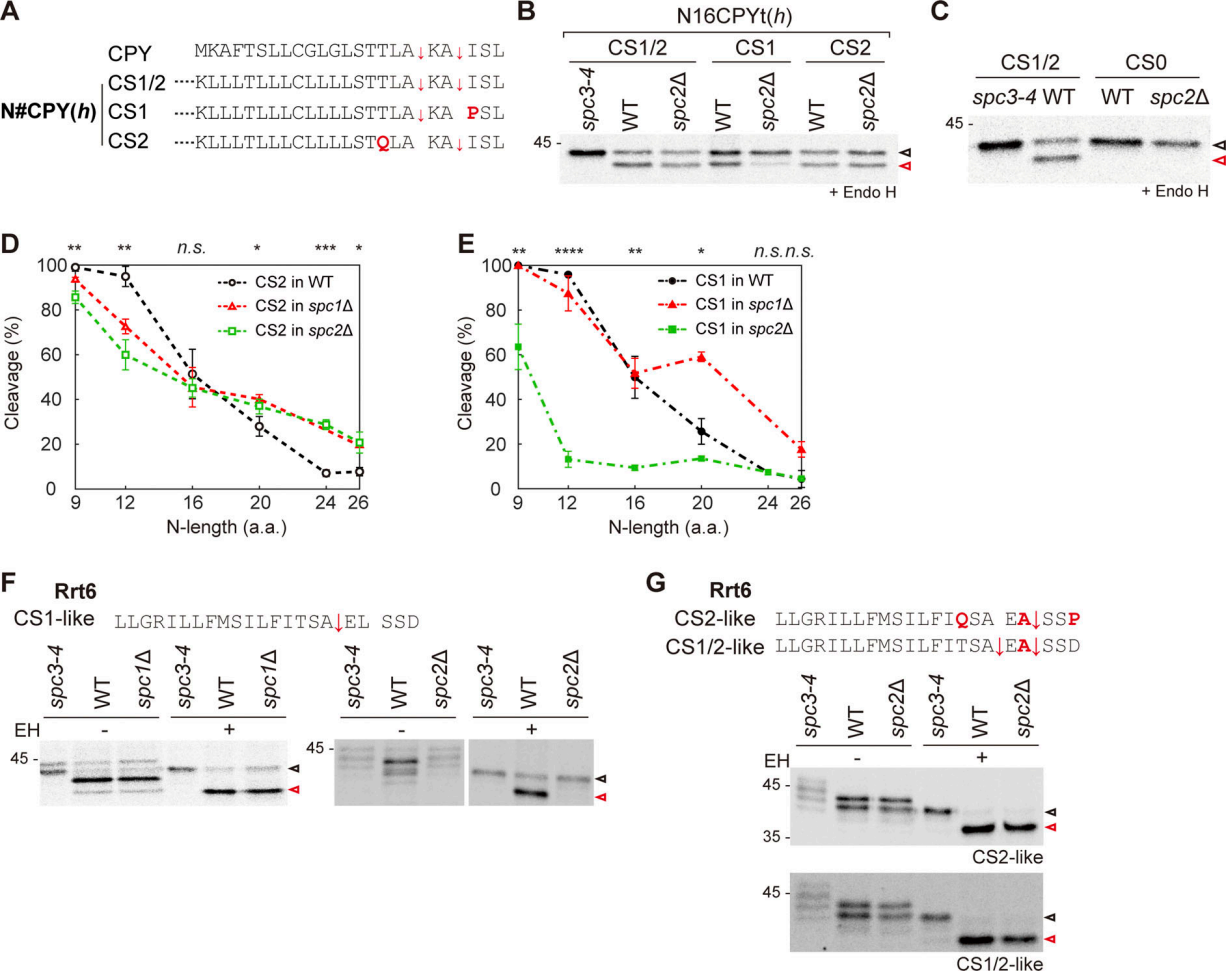
**Recognition of signal sequence cleavage sites in the *spc2*Δ strain. (A)** Signal sequences of CPYt and N#CPYt(*h*) cleavage site (CS) variants. **(B)** N16CPYt(*h*) CS1/2, CS1 and CS2 in the *spc3-4*, WT, and *spc2*Δ strains were pulse-labeled and subjected to Endo H treatment prior to SDS-PAGE. **(C)** N16CPYt(*h*) CS1/2 and CS0 in the *spc3-4* and WT strains were assessed by pulse labeling. **(D)** Cleavage efficiencies of N#CPYt(*h*) CS2 variants in the *spc2*Δ strain (green). **(E)** CS2 variants in the *spc2*Δ strain (green). Cleavage profiles of CS2 or CS1 variants in the *spc1*Δ strain (red) (Yim et al., 2021) are shown for comparison. Three independent experiments were carried out (*n* = 3/data point), and the average is shown with the standard deviation. P values between CS1 variants in the WT and *spc2*Δ, and between CS2 variants in the WT and *spc2*Δ were calculated by multiple two-tailed *t* tests; n.s., P > 0.05; *, P ≤ 0.05; **, P ≤ 0.01; ***, P ≤ 0.001; ****, P ≤ 0.0001. **(F and G)** Signal sequences and the downstream residues of Rrt6 (F) and its CS variants (G). Mutated residues are colored in red, and potential cleavage sites are indicated with an arrow (↓). The indicated Rrt6 CS variants in the *spc3-4*, WT, and *spc2*Δ strains were analyzed by pulse labeling. A representative of at least three experiments is shown. De-glycosylated full-length and cleaved products are indicated in unfilled black and red arrows, respectively. Source data are available for this figure: SourceData F4.

CPY contains two potential cleavage sites (CS1 and CS2), CS1 is located proximal to and CS2 is distal to the *h*-region (Fig. 4 A and Fig. S2 C), thus all its variants also contain them. In WT cells, CPY is cleaved at CS2 (Blachly-Dyson and Stevens, 1987), but the “cryptic” CS1 is efficiently cleaved when CS2 is mutated (Yim et al., 2021). To assess whether cleavage site selection is influenced by Spc2, each site was inactivated by mutagenesis. We first assessed the processing of N16CPYt(*h*) CS variants in WT and *spc2*Δ strains and found that inactivation of CS1 (CS2 variant) had no effect on the cleavage in either strain, but inactivation of CS2 (CS1 variant) dramatically decreased cleavage in the *spc2*Δ strain (Fig. 4 B). When both cleavage sites were mutated, no processing was seen in either the WT or the *spc2*Δ strain (Fig. 4 C).

Next, we assessed the cleavage of other N#CPYt(*h*) variants having only CS1 or CS2. Cleavage profiles of CS2 N#CPYt(*h*) variants in the WT and *spc2*Δ strains were similar to those of CS1/2 N#CPYt(*h*) variants (Fig. 4 D). However, cleavage of CS1 N#CPYt(*h*) variants was dramatically decreased to <20% in *spc2*Δ cells, except for N9CPYt(*h*), which showed ∼60% cleavage (Fig. 4 E). In comparison, as observed previously (Yim et al., 2021), both CS1 and CS2 variants were efficiently cleaved in *spc1*Δ cells (Fig. 4, D and E). These data indicate that CS1 can be efficiently cleaved by SPC or by SPC lacking Spc1, but that it is poorly recognized in the absence of Spc2.

We further checked the cleavage of Rrt6, a protein that mediates vesicle transport between the ER and Golgi (Hirata et al., 2013). Rrt6 has an SP with multiple predicted cleavage sites in the *c*-region; the proximal CS1-like site has the highest prediction score (Fig. S2 D). Wildtype Rrt6 was cleaved in the WT and *spc1*Δ strains; however, the cleavage was completely inhibited in the *spc2*Δ strain (Fig. 4 F). A few substitutions were introduced to convert the Rrt6 SP into CS2-like and CS1/2-like variants (Fig. S2 D), and their cleavage was assessed. Both were efficiently processed in the WT and *spc2*Δ strains (Fig. 4 G). These data showed that the cleavage site proximal to the *h*-region is less efficiently recognized by SPC lacking Spc2, similar to the CS1 N#CPYt(*h*) variants, suggesting that Spc2 (but not Spc1) plays a key role in flexible recognition of the cleavage site by SPC. In turn, these results imply that substrate selection and cleavage by SPC is an intricate process that depends on features of the *n*-, *h*-, and *c*-regions and that Spc1 and Spc2 have distinct effects on SP cleavage site selection and cleavage efficiency.

### Coarse-grained MD-simulation of yeast SPC models with and without Spc2

**Figure S3.**
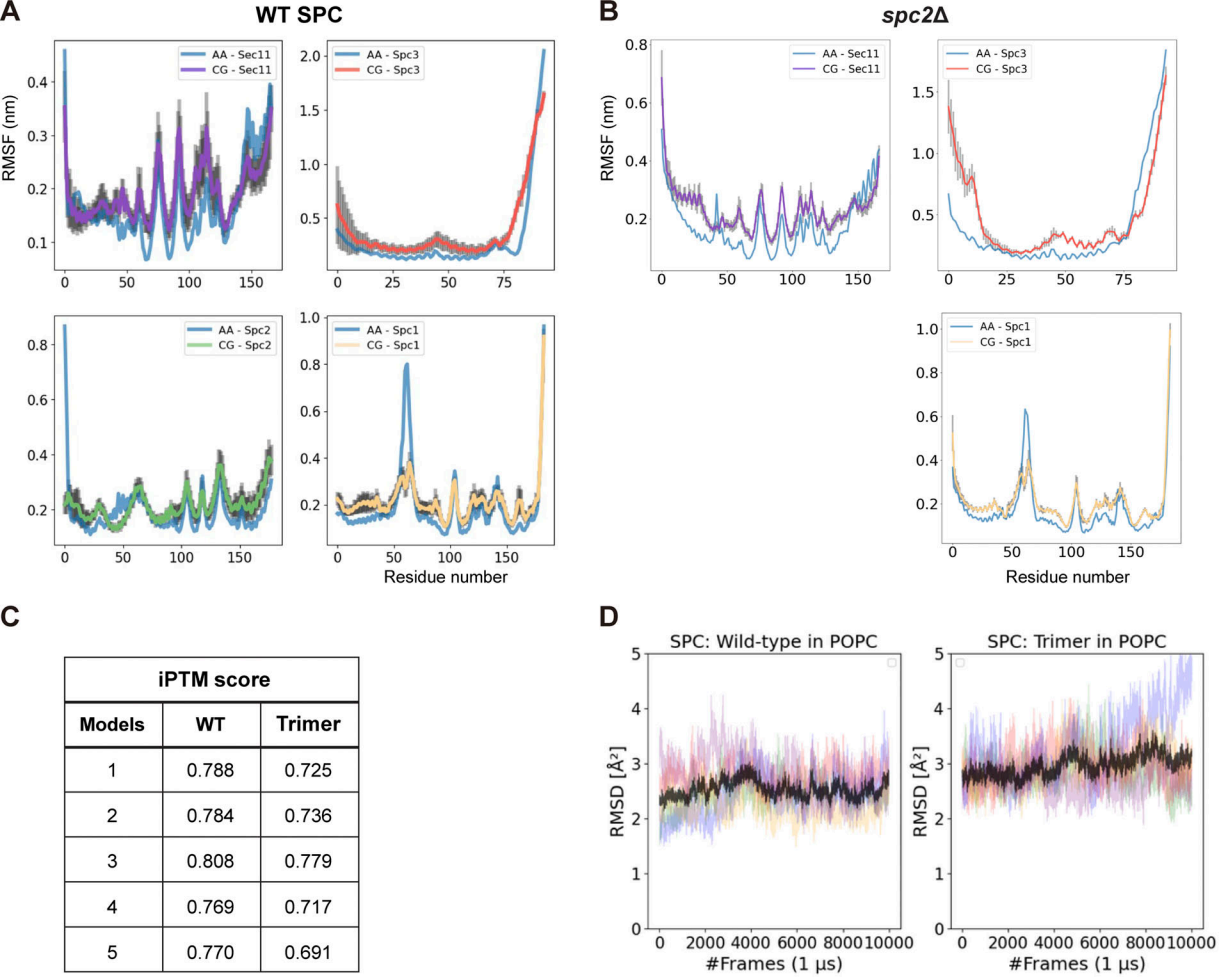
**Provides model validation for the MD simulations. (A and B)** Comparison of the α-carbon root mean squared fluctuations (RMSFs) between all-atom (AA) and coarse-grained (CG) MD simulations in a POPC bilayer, per subunit. **(A)** WT SPC and (B) SPC lacking Spc2 (spc2Δ). Sec11 is shown in purple, Spc3 in red, Spc1 in orange, Spc2 in green, the atomistic reference RMSF is colored in blue and error bars are shown in grey. **(C)** iPTM scores for each model predicted by AlphaFold2-Multimer (V3) for the tetramer and trimer (spc2Δ) SPC. **(D)** RMSD time-series extracted from all-atom MD simulations of the wild-type and trimeric variants of yeast SPC embedded in a POPC bilayer. The different colors correspond to individual 1 μs long simulation runs and the black line is an average RMSD computed from the five repeats.

**Figure 5.**
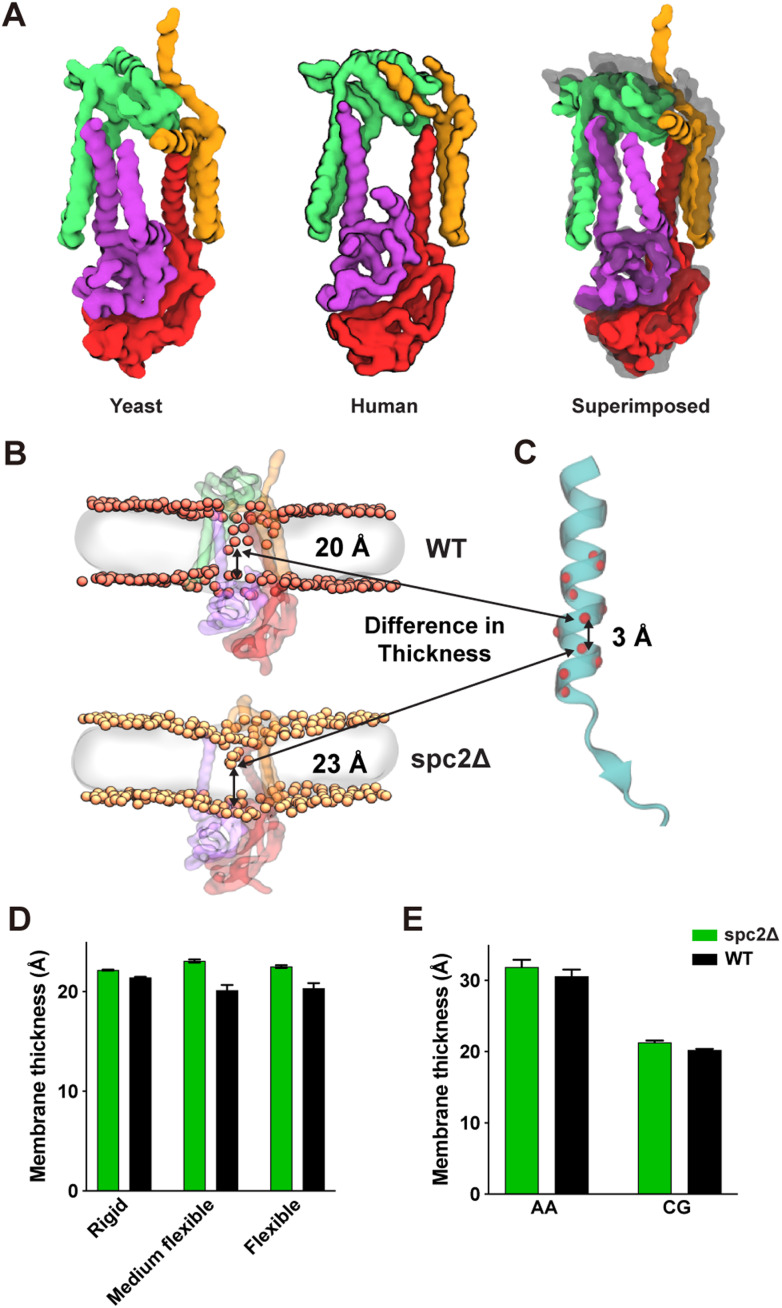
**AlphaFold2 predictions and MD simulations of the yeast SPC with and without Spc2. (A)** Structural alignment of the AF2-predicted yeast SPC with the cryo-EM human SPC (Liaci et al., 2021). Spc2 in green, Sec11 in purple, Spc3 in red, Spc1 in orange. **(B)** Representative snapshot of the TM window membrane thickness for SPC with (top, WT) and without Spc2 (bottom, spc2Δ). The phosphate headgroups are colored in salmon (WT) or yellow (spc2Δ), and the lipid tails are represented as a transparent grey surface. **(C)** Model peptide illustrating that the difference in membrane thickness qualitatively fits with a hypothetical SPC substrate selectivity filter for SAs and SPs. **(D)** Membrane thickness computed for the ER membrane-embedded SPC using different protein models: a rigid model, elastic network models; a semi-flexible and fully flexible model. **(E)** Membrane thickness computed for a system composed of the yeast SPC embedded into a POPC membrane at atomistic or Martini 3 resolution.

CGMD simulations of the human SPC cryo-EM structure using the Martini 3 coarse-grained model (Souza et al., 2021) show that the membrane region where the SPC binds SPs (the “TM window”) is significantly thinner than the bulk membrane (Liaci et al., 2021). To better understand how the yeast SPC with and without Spc2 behave in the membrane, CGMD simulations were carried out on a yeast SPC model predicted by AlphaFold2-Multimer (Evans et al., 2022, *Preprint*) inserted into a model of the yeast ER membrane (Monje-Galvan and Klauda, 2015). Model validation was carried out by comparing the root mean squared fluctuation (RMSF) of the backbone beads to the Cα RMSF obtained using atomistic simulations of SPC with and without Spc2 inserted into a POPC bilayer (Fig. S3, A and B). Furthermore, we evaluated protein structure confidence and structural integrity by extracting the AlphaFold score for all five predicted models (Fig. S3 C) and, for the top-ranked model, the root mean squared deviation (RMSD) time series was computed from five 1-μs long all-atom MD simulations (Fig. S3 D). The predicted yeast SPC structure agrees well with the human SPC structure from Liaci et al. (2021) (Fig. 5 A).

**Figure S4.**
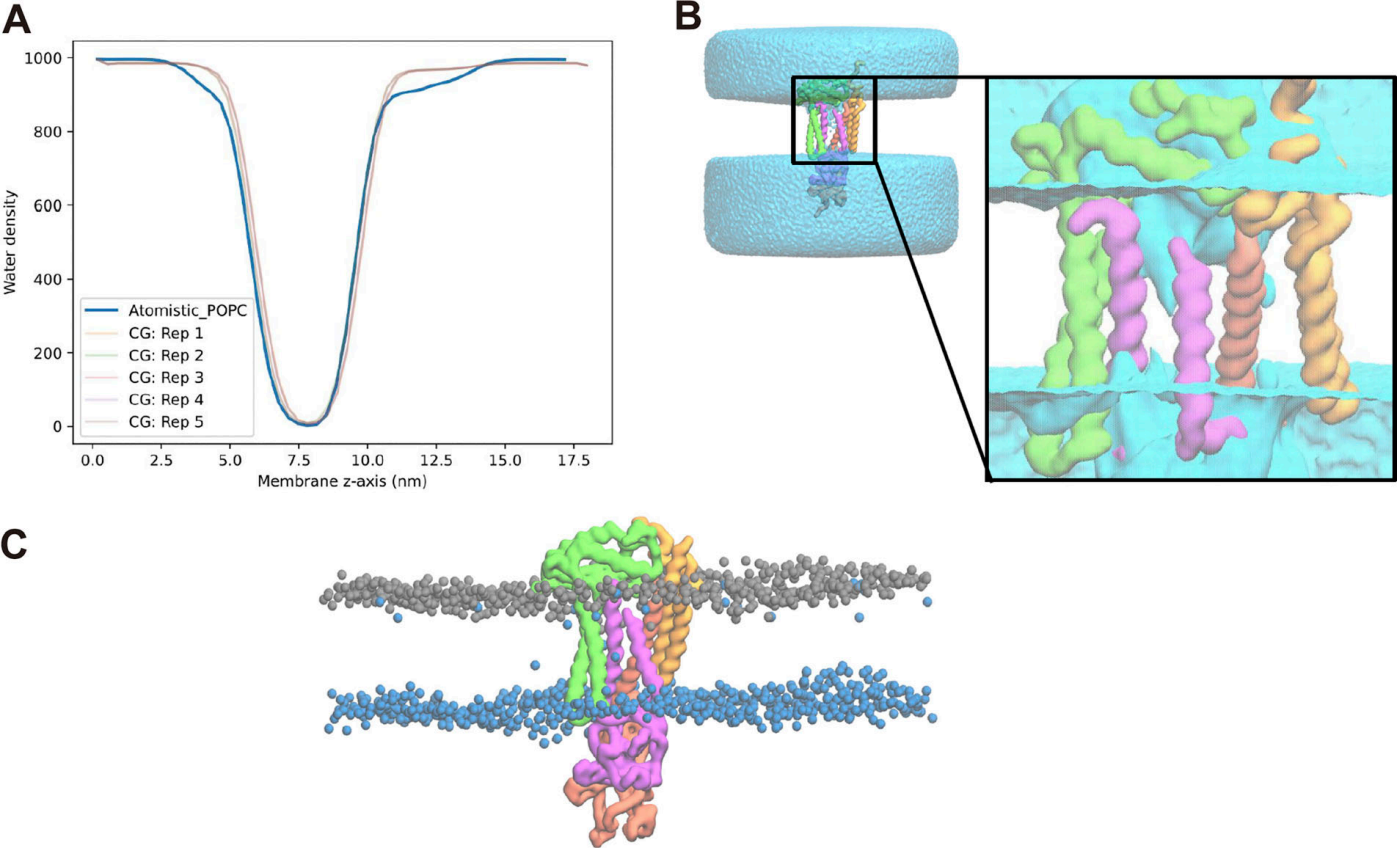
**Includes MD simulation data on water density profiles and maps. (A)** Water density profiles computed using gmx density. The solid line is computed from an all-atom MD simulation with SPC embedded in a POPC bilayer. The faded lines correspond to water densities extracted from five independent 20 μs MD simulations of SPC embedded in a model endoplasmic reticulum membrane. **(B)** Snapshot illustrating water penetration inside the SPC window without the formation of a water pore. **(C)** Snapshot showcasing a degree of lipid flip-flop across the ER membrane model.

**Figure S5.**
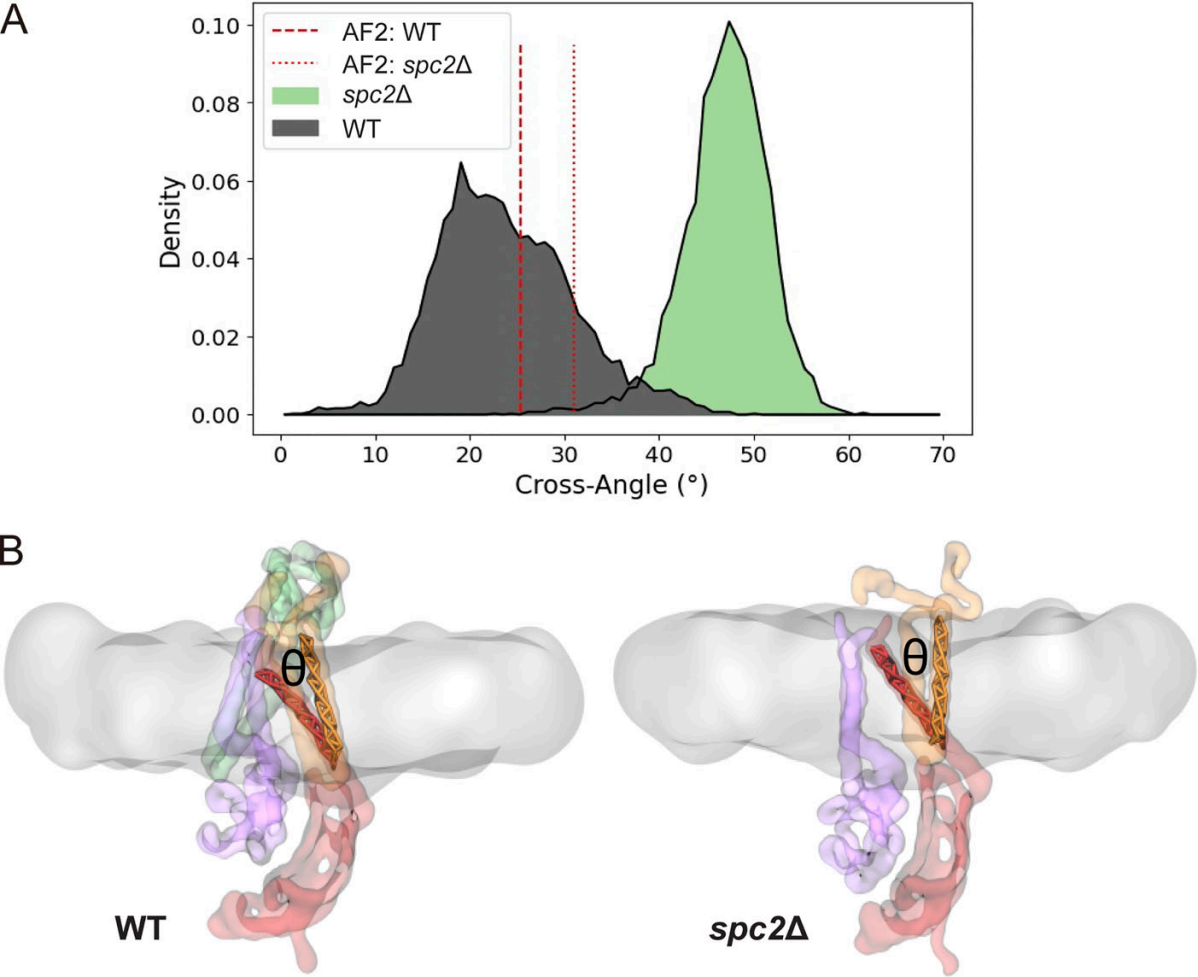
**Illustrates structural rearrangements of the SPC upon removal of Spc2. (A)** Crossing angle computed from the vector defined in the Spc3 TM helix 1, between residues 4 and 23, and the vector defined in the Spc1 TM helix 1, between residues 20 and 43. Single-point cross-angles calculated from the AF2 structures are shown in red vertical lines. **(B)** Representative snapshots illustrating the SPC WT (left) and *spc2*Δ (right) structures. The phosphate headgroups are colored in salmon (WT) or yellow (*spc2*Δ), Spc2 in green, Sec11 in purple, Spc3 in red, Spc1 in orange, and the lipid tails are represented as a transparent grey surface.

Compared with the bulk ER membrane thickness (38.1 ± 0.01 Å), the membrane region in the TM window was ∼47% thinner (20.1 ± 0.54 Å) whereas the membrane in the same region in the absence of Spc2, which corresponds to a trimeric form (spc2Δ), was only ∼40% thinner than bulk membrane (23.1 ± 0.16 Å) (Fig. 5, B and D). The difference in membrane thickness between SPC with and without Spc2 corresponds to ∼7–10%, which translates into a 3.0 ± 0.7 Å difference (equivalent to two to three residues in an α-helix, Fig. 5 C). The same trend was observed for systems employing a POPC membrane both at the atomistic and CG resolutions (Fig. 5 E). We also observed that in the wild-type variant, there is a significant amount of water that could penetrate the TM window. While not forming a pore (Fig. S4), the water density there may also contribute to the membrane thinning by increasing the polarity of the environment around the TM window. Removal of Spc2 also induces structural changes in the SPC, leading to an adaptation of the TM window where Spc3 tilts downward and Spc1 tilts upward, such that the topmost part of the SPC TM window is covered, as observed by the cross-angle between the large helix from Spc3 (red) and TM1 of Spc1 (orange), Fig. S5.

### Effects of polar residues in Spc2 TM on membrane thickness

**Figure 6.**
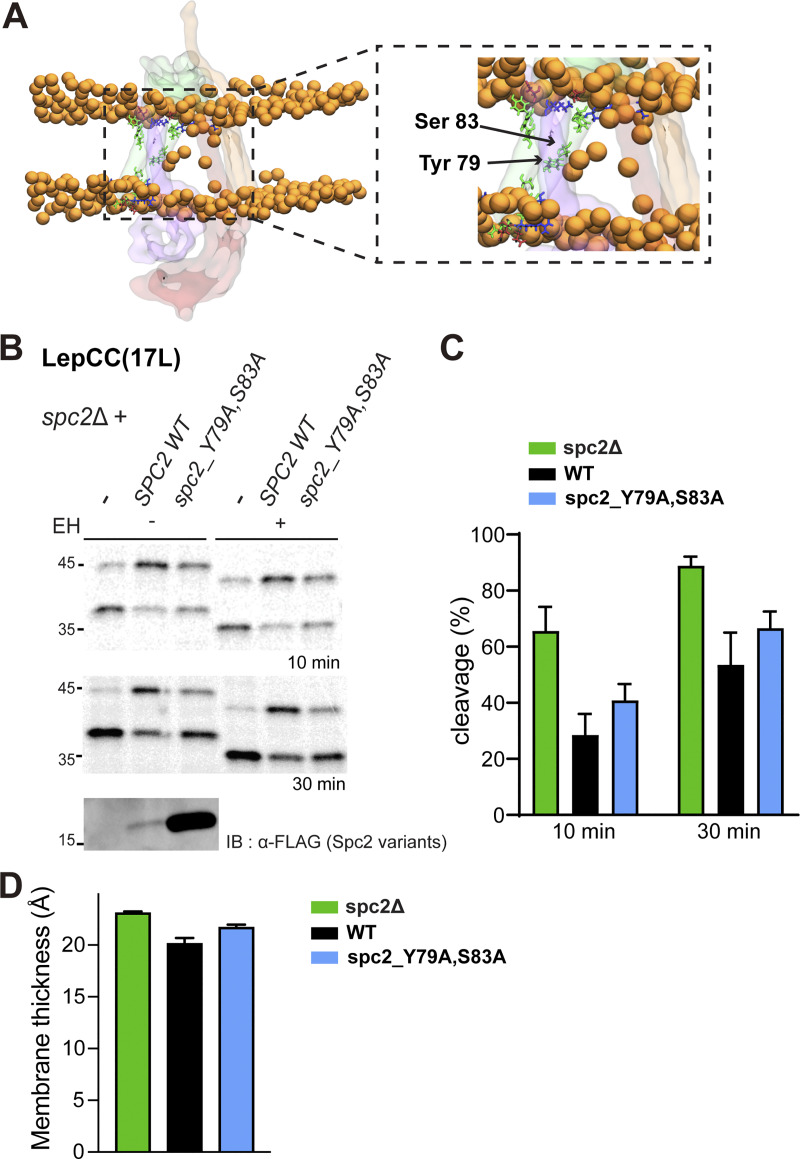
**Effects of polar residues in Spc2 TM on membrane thickness. (A)** Localization of the polar (green) and charged (blue for negative, red for positive) residues of Spc2 within the TM window allow for deeper-lying phosphate headgroups within the membrane. **(B)** LepCC(17L) in *spc2*Δ cells with an empty vector (−), *SPC2*, *spc2-Y79A*, *S83A* were analyzed by pulse labeling for 10 and 30 min. Bottom: Expression of Spc2 (under its endogenous promoter in the CEN plasmid) and *spc2-Y79A*,*S83A* (under the GPD promoter in 2 µm plasmid) is shown. **(C)** Cleavage (%) was quantified and plotted as in Fig. 6 B. Three independent experiments were carried out, and the average is shown with the standard deviation. **(D)** Membrane thickness in the TM window for yeast SPC without Spc2 (spc2Δ), with Spc2 (WT) and with a double mutated variant of Spc2 (spc2_Y79A, S83A), embedded in a model of the yeast ER membrane, computed from Martini 3 CGMD simulations. Average values across five 20 μs simulations per system for spc2Δ and WT, and four 4 μs simulations for spc2_Y79A, S83A are shown with the standard error of the mean. Source data are available for this figure: SourceData F6.

Spc2 possesses several polar or charged residues, such as Tyr79 and Ser83, within its TM helices, which can coordinate phosphate headgroups deep within the TM window in CGMD simulations (Fig. 6 A). In the absence of Spc2, we observed that some of the deeper-lying phosphates are no longer inside the TM window, leading to an increase in membrane thickness (Fig. 5 B). To experimentally assess the effects of polar residues in Spc2 on SP cleavage, LepCC(17L) cleavage was assessed in *spc2*Δ cells carrying *spc2_Y79A*,*S83A*, where two polar residues in Spc2 TM2 were mutated to Ala (Fig. 6, B and C). LepCC(17L) was more efficiently cleaved in the presence of spc2_Y79A,S83A than in the presence of Spc2, although the effect was smaller than in the absence of Spc2. These data suggest that polar residues in Spc2 TM seen in the CGMD simulations indeed contribute to the thinning of the TM window. This was further confirmed by carrying out CGMD simulations with the spc2_Y79A,S83A double mutant, where we observed that the mutations led to a thickening of the membrane within the TM window to around 22 Å (Fig. 6 D).

Our data thus suggest that Spc2 is critical for proper thinning of the membrane and thereby prevents access of signal sequences with longer *h*-regions, such as found in LepCC(17L), to the central TM window, preventing their cleavage by SPC. The 14-residue long TM helix in LepCC(14L) would fit in a ∼21 Å thick membrane, whereas the 17-residue long TM helix in LepCC(17L) would require a ∼24 Å thick membrane, in good agreement with the simulation data, Fig. 5 B. Overall, these data suggest that one function of Spc2 may be to modulate the membrane environment in and around the SPC to improve its ability to discriminate between SPs and SAs.

## Discussion

**Figure 7.**
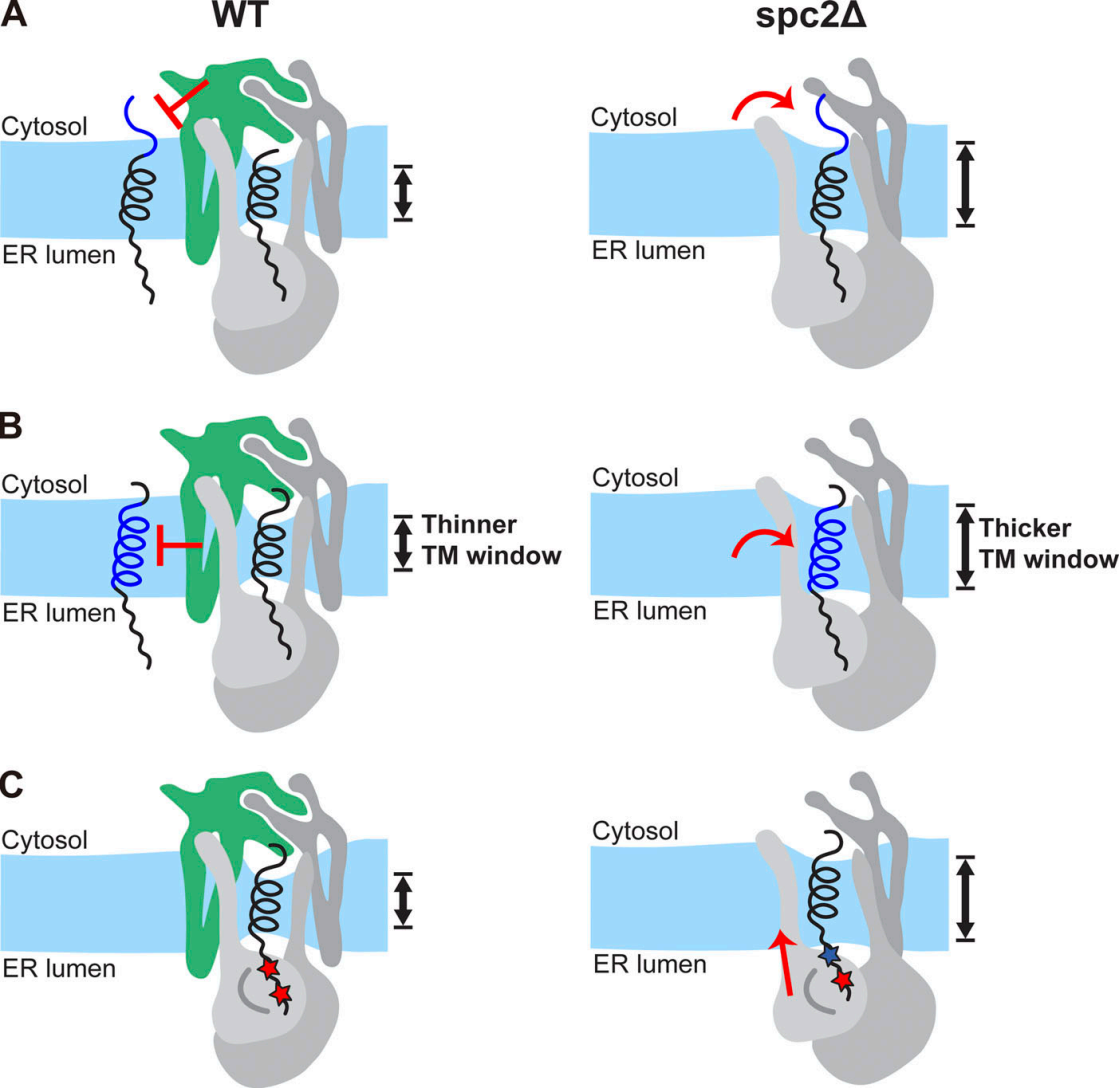
**Proposed models for Spc2-mediated substrate and cleavage site selection. (A and B)** The cytosolic domain of Spc2 prevents access of signal sequences with longer *n*-regions to the TM-window, (B) membrane thinning induced by Spc2 prevents access to signal sequences with longer *h*-regions to the TM-window. **(C)** Membrane thinning induced by Spc2 increases the exposure of proximal cleavage sites to the SPC active site (the blue star indicates a proximal cleavage site rendered inaccessible by the absence of Spc2).

Our data provide insights into the underlying mechanism of how SP and SA sequences are distinguished by the SPC. First, we observed that signal sequence recognition and cleavage depend on *n*-region length. For that, the C-terminal domain of Spc2 is particularly critical (Fig. 2). This domain covers the cytosolic side of the SPC (Liaci et al., 2021) and may thus sterically prevent signal sequences with long *n*-regions from entering the TM-window. This may be the reason why SPs with short *n*-regions are preferable substrates for the SPC (Fig. 7 A).

Second, CGMD simulations of the yeast SPC structure predicted by AlphaFold2-Multimer show that the membrane within the TM-window is thinner compared with the bulk membrane thickness, as observed also for the human SPC cryo-EM structure (Liaci et al., 2021) (Fig. 5). Thus, SPs with shorter *h*-regions would fit in the thinned membrane whereas SAs with longer *h*-regions would be too long to fit, thereby preventing access to the SPC active site. We further observe that the cleavage of model signal sequences with relatively long *h*-regions is markedly increased in the absence of Spc2 (Fig. 3). These results are in agreement with data from the CGMD simulations of the yeast SPC structure without Spc2, in which the membrane is ∼3 Å thicker in the TM-window compared with the structure with Spc2 (Fig. 5). This difference in thinning is similar to the difference in length between α-helices composed of 15 and 17 residues. These data suggest that substrates with longer *h*-regions may only fit in the thicker TM-window present in SPC lacking Spc2, but are discriminated against in the intact SPC (Fig. 7 B). We noted that membrane thinning may be important also for prokaryotic signal peptidases such as the well-studied signal peptidase I (LepB; Uniprot ID P00803) from *Eschericiha coli*. LepB is anchored in the inner membrane by two N-terminal TMs, one of which has a hydrophobic segment that is only ∼15 residues long and is bracketed by charged residues, thus potentially inducing local thinning in an area of the membrane located close to the LepB active site.

Third, the cleavage site of the signal sequences needs to be exposed to the luminal side of the membrane to reach the catalytic site of SPC. By mutating one of two potential cleavage sites at a time in N#CPYt(h) variants, we observed that both sites were efficiently used in WT cells, indicating that both sites are within reach of the catalytic site of SPC (Fig. 4). However, the cleavage efficiency of the cleavage site proximal to the *h*-region was significantly reduced when Spc2 was absent. Although further studies are needed for a full understanding of cleavage-site selection, we speculate that the cleavage site proximal to the *h*-region may be occluded or pulled into the less thinned membrane in the TM-window in *spc2*Δ cells, preventing cleavage (Fig. 7 C).

### The impact of Spc2 on proteostasis

Previous studies have shown that the unfolded protein response (UPR) in the ER upregulates gene expression of Spc2 (but not Spc1) (Travers et al., 2000) and deletion of Spc2 triggers the UPR in the ER (Jonikas et al., 2009), identifying Spc2 as a crucial component in protein folding in the ER. Indeed, we observed that Kar2 and Pdi1, two ER chaperones were upregulated upon deletion of Spc2. However, it has been unclear how Spc2 affects protein folding in the ER since its deletion did not affect the cleavage of the few secretory proteins tested before. Our results suggest that Spc2 particularly affects discrimination and cleavage of secretory precursors with suboptimal SP features (i.e., unusually long *n*- or *h*-region, proximal cleavage site). In turn, this is likely to affect the folding, maturation, and localization of those abnormally processed or unprocessed secretory proteins, impacting ER proteostasis. In line with the role of yeast SPC in proteostasis, a recent study shows that human SPC acts as a quality control enzyme for membrane proteins (Zanotti et al., 2022).

## Materials and methods

### Yeast strains

The *S*. *cerevisiae* haploid W303-1α (*MATα*, *ade2*, *can1*, *his3*, *leu2*, *trp1*, *ura3*) was used as a WT strain. The *SPC2* ORF in W303-1α was replaced with the *HIS3* gene amplified from the pCgH plasmid (Kitada et al., 1995) by homologous recombination to generate the *spc2*Δ strain (*MATα*, *ade2*, *can1*, *his3*, *leu2*, *trp1*, *ura3*, *SPC2*:*:HIS3*). The 3′ end of the *SPC3* ORF in the *spc2*Δ strain was tagged with the triple HA sequence amplified from the pFA6a-3HA-KANMX6 plasmid (39295; Addgene) by homologous recombination to generate the *spc2*Δ,*SPC3HA* strain (*MATα*, *ade2*, *can1*, *his3*, *leu2*, *trp1*, *ura3*, *SPC2*:*:HIS3*, *SPC3-3HA-KANMX6*). *Spc3-4* is an SPC catalytic mutant (Fang et al., 1997).

### Construction of plasmids

Open reading frame (ORF) of *ECM38*, *KAR2*, *PHO8*, *SPC2*, and *RRT6* were PCR-amplified using the isolated genomic DNA of *S*. *cerevisiae* as a PCR template with the following primers: 5′-CGG​ATT​CTA​GAA​CTA​GTG​GAT​CCA​TGC​TGT​TGT​GTA​ATA​GAA​AAG​TCC-3′ and 5′-GTA​TGG​GTA​AGA​TGG​CTG​CAG​GTA​TAC​GGA​GGA​GAT​TCC​TCT​TTT​TC-3′ for ECM38; 5′-CGG​ATT​CTA​GAA​CTA​GTG​GAT​CCA​TGT​TTT​TCA​ACA​GAC​TAA​GCG​C-3′ and 5′-GTA​TGG​GTA​AGA​TGG​CTG​CAG​CAA​TTC​GTC​GTG​TTC​GAA​ATA​ATC-3′ for KAR2; 5′-CGG​ATT​CTA​GAA​CTA​GTG​GAT​CCA​TGA​TGA​CTC​ACA​CAT​TAC​CAA​G-3′ and 5′-GTA​TGG​GTA​AGA​TGG​CTG​CAG​GTT​GGT​CAA​CTC​ATG​GTA​GTA​TTC-3′ for PHO8; 5′-CGG​ATT​CTA​GAA​CTA​GTG​GAT​CCA​TGA​GTT​CTG​CTA​AAC​CTA​TTA​ATG​TAT​ATT​C-3′ and 5′-CTT​ATC​GTC​GTC​ATC​CTT​GTA​ATC​TTC​ATT​TTT​TTT​GGT​GTC​GAG​GAC-3′ for SPC2; 5′-CGG​ATT​CTA​GAA​CTA​GTG​GAT​CCA​TGG​AAA​AAG​CTT​CCT​TGA​ACA​T-3′ and 5′-GTA​TGG​GTA​AGA​TGG​CTG​CAG​CAA​ACC​TTT​CTT​TTT​GAT​ATG​AGA​TGA​AG-3′ for RRT6. Gene fragments were inserted into pRS424GPD (*ECM38*, *KAR2*, *PHO8*, and *RRT6*) or pRS426GPD (*SPC2*) vectors (Mumberg et al., 1995) having the triple HA or FLAG sequence for the C-terminal tagging via Gibson Assembly. Site-directed mutagenesis was carried out on these plasmids to construct Pho8(P54A), Spc2-ΔCD, Spc2-TM2*, CS2-like and CS1/2-like Rrt6, and Spc2_Y79A,S83A following the manufacturer’s protocol (KOD-Plus-Mutagenesis Kit; Toyobo). For the construction of plasmid expressing *SPC2* under endogenous promoter, *SPC2* including the sequence 1.5 kb upstream and 0.68 kb downstream was PCR-amplified with the primers 5′-CGC​TCT​AGA​ACT​AGT​GGA​TCC​CCT​GGA​CAC​TTA​CCG​TCT​TC-3′ and 5′-GCT​TGA​TAT​CGA​ATT​CCT​GCA​GCG​AAG​ATG​TTA​TCA​AAG​CAG​CAG-3′ and inserted into pRS416 vector (GenBank U03450) via Gibson Assembly. Then, *SPC2* was C-terminally tagged with FLAG by site-directed mutagenesis. Plasmids encoding *CPY* and LepCC variants were previously constructed as in Yim et al. (2021).

### Pulse-labeling and immunoprecipitation

Yeast cells were grown in a synthetic complete medium until an optical density (OD_600_) reached between 0.3 and 0.8. Then, 1.5 OD unit cells were harvested at 2,000 *g* for 5 min at 4°C, washed once with minimal media (-Met, -ammonium sulfate), and preincubated at 30°C for 10–15 min before pulse labeling. Met-starved cells were then radiolabeled with [^35^S]-Met (40 μCi per 1.5 OD_600_ units of cells) in 150 μl of −Met medium for 5 min except that Kar2 was pulse-labeled for 3–5 min and LepCC(17L) in Fig. 6 B for 10 and 30 min at 30°C. For experiments with *spc3-4* strain (Fang et al., 1997), cells were starved and radiolabeled at 24°C or 37°C. Ice-cold buffer containing 20 mM sodium azide was added to stop labeling and cells were spun down and stored at −20°C until use. Cells were resuspended in lysis buffer (20 mM Tris-HCl, pH 7.5, 1% SDS, 1 mM DTT, 1 mM PMSF, and 1x Protease Inhibitor Cocktail [QTPPI1011; Quartett]) and mixed with 100 μl of ice-cold glass beads. Cell suspensions were vortexed for 2 min twice, keeping the samples on ice for 1 min in between. The cell lysate was then incubated at 60°C for 15 min and spun down at 6,000 *g* for 1 min at 4°C to remove cell debris. The supernatant was transferred to a fresh tube and mixed with 500 μl of IP buffer (15 mM Tris-HCl pH 7.5, 0.1% SDS, 1% Triton X-100, and 150 mM NaCl), 20 μl of prewashed 40% slurry Protein G-Agarose (Cat# 20397; Thermo Fisher Scientific; Pierce), and 1 μl of anti-HA antibody (Cat# MMS-101R; Biolegend). Samples were rotated for 3 h at room temperature. After 3 h of incubation, the IP-agarose beads were spun down and washed twice with IP buffer, once with ConA buffer (500 mM NaCl, 20 mM Tris-HCl pH 7.5, and 1% Triton X-100), and once with Buffer C (50 mM NaCl and 10 mM Tris-HCl pH 7.5), respectively. For all washing steps, the tubes were spun down for 30 s to settle down agarose beads. The IP sample was resuspended in sample buffer (50 mM DTT, 50 mM Tris-Cl pH 7.6, 5% SDS, 5% glycerol, 50 mM EDTA pH 8, 1 mM PMSF, 1x Protease Inhibitor Cocktail [QTPPI1011; Quartett], and bromophenol blue) and incubated at 60°C for 15 min. Samples were incubated with Endo H (Cat# V4871; Promega or Cat# P0703L; NEB) at 37°C for 30–60 min.

### Western blot analysis

Proteins were prepared either by rapid protein preparation or trichloroacetic acid (TCA) precipitation. For rapid protein preparation, ∼5–10 OD_600_ units of cells were harvested, washed once with 5 ml of distilled water, and resuspended with 50–100 μl of SDS sample buffer. The supernatant was obtained by centrifugation at 20,000 *g* for 5 min at room temperature, transferred to a new tube, and incubated at 95°C for 5–10 min before loading on a SDS-gel. TCA precipitation of proteins was done by adding a final concentration of 25% TCA to the cell lysate. The precipitated protein pellet was washed with 500 μl of acetone, resuspended in 50–100 μl of sample buffer, and incubated at 95°C for 10 min. Samples were treated with Endo H (Cat# P0703L; NEB) at 37°C for 30 min. Prepared protein samples were subjected to SDS‒PAGE and western blotting with anti-HA (Cat# 901513; Biolegend), anti-FLAG (Cat# 200-301-383; Rockland), and anti-Pgk1 (Cat# 113687; Abcam) antibodies. Bands were detected using Lumigen ECL Ultra (Cat# TMA-6; Lumigen) by ChemiDoc XRS+ (Bio-Rad), and the resulting data were processed using Image Lab (Bio-Rad) software.

### Autoradiography and quantification of bands

Typhoon FLA 7000 or FLA 9500 phosphoimager (GE Healthcare) was used for the detection of radiolabeled bands on SDS-gels. Imaging data were processed, and the detected bands were quantified using MultiGauge version 3.0 software. Cleavage efficiency was calculated from the band intensities of glycosylated bands using the formula: cleavage (%)=cleaved band×100/(cleaved+full−length bands).

### Statistical analysis

Statistical analysis of data was performed using Microsoft Excel 2013 (RRID:SCR016137) (Microsoft), MATLAB, or GraphPad Prism 10. Data distribution was assumed to be normal but this was not formally tested. In Fig. 3 E, unpaired *t* test with Welch’s correction was performed using GraphPad Prism 10 and the two-tailed P value was calculated.

### Quantitative mass spectrometry analysis

W303-1α and *spc2*Δ cells were grown in SC (Synthetic Complete) medium, and *spc2*Δ cells carrying *SPC2*, *spc2-ΔCD*(*58*), or spc2-TM2* in synthetic drop out (−Ura) medium overnight at 30°C in three biological replicates. Thirty OD_600_ units of cells were harvested, resuspended in 250 μl of lysis buffer (8 M urea, 50 mM HEPES, pH 8.5, 75 mM NaCl, 0.1 mM PMSF, 1x Halt Protease Inhibitor Cocktail (Thermo Fisher Scientific), and 5 mM EDTA), mixed with glass beads, and subjected to vortexing for 6 min to 10 min. The resulting cell lysates were reduced with 5 mM dithiothreitol (DTT) at 37°C, alkylated with 10 mM iodoacetamide (IAA) in the dark for 1 h, and quenched with 15 mM DTT. After dilution of lysis buffer with 50 mM HEPES, pH 8.5, to reduce the urea concentration below 1 M, protein samples were digested with trypsin or sequential digestion with LysC and trypsin overnight. Trypsin-digested samples were desalted using SOLA reversed-phase polymeric cartridges (Thermo Fisher Scientific) and labeled with TMT-11 plex labeling reagents (Pierce). After again desalting, pooled TMT-labeled peptides were subjected to LC‒MS/MS.

LC‒MS/MS analysis was performed using an UltiMate 3000 RSLCnano system (Thermo Fisher Scientific) coupled to an Exploris 480 mass spectrometer (Thermo Fisher Scientific). Samples were analyzed on an Acclaim PepMap C18 100 column (0.075 × 500 mm, 3 µm particle size) with a 240-min gradient. Exploris 480 was operated with a spray voltage of 2 kV and an ion source temperature of 325°C in positive ion mode. MS1 spectra were collected at 120,000 resolution at a range of 400–1,400 m/z. MS/MS spectra were acquired at a resolution of 45,000 with the fragmentation of the top 20 peaks and a dynamic exclusion window of 45 s.

Raw LC‒MS/MS data files were processed using Proteome Discoverer 2.4 (Thermo Fisher Scientific). A precursor mass tolerance of 10 ppm and fragment mass tolerance of 0.02 Da were used with methionine oxidation and protein N-terminal acetylation set as variable modifications, and TMT6plex (+229.163 Da) at peptide N-termini and lysine residues and carbamidomethylation of cysteines were set as static modifications. High-confidence peptides were validated with a target FDR set to 0.01. Protein identifications of high confidence were determined with an FDR of 0.01 and quantified based on at least one high-confident peptide.

### Structure prediction and preparation for MD simulations

For CGMD simulations, the starting structures were predicted using AlphaFold2-Multimer (Evans et al., 2022, *Preprint*). The WT SPC model was validated by comparing it to the human SPC structure (Liaci et al., 2021). The protonation state of the titratable residues was computed for the WT variant using PROPKA 3.0 (Jurrus et al., 2018; Olsson et al., 2011; Søndergaard et al., 2011) at pH 7.0 and the recommended protonation states were retained. For SPC lacking Spc2, the same protonation state from the WT SPC was used.

### All-atom MD simulations of yeast SPC

To carry out atomistic simulations of the WT and *spc2*Δ variants of SPC, the proteins were first embedded into a POPC bilayer in a 15 × 15 × 15 cubic box and then solvated in TIP3P water (Jorgensen et al., 1983) and 0.15 M of NaCl using CHARMM-GUI (Brooks et al., 2009; Jo et al., 2008; Lee et al., 2016). The principal axis of each SPC variant and the normal of the bilayer were then aligned to the z-axis using OPM (Lomize et al., 2006) within CHARMM-GUI. One simulation per variant was carried out and was later used to compare protein flexibility between atomistic and CG resolutions by evaluating their alpha-carbon root mean squared fluctuations.

The simulation boxes were first energy minimized for 5,000 steps using the steepest descent, enforcing position restraints to the backbone, sidechains, and lipids (4,000, 2,000, and 1,000 kJ/mol). After system minimization, six steps of relaxation at 310 K and 1 bar pressure were carried out where the position restraints were gradually removed. The first three relaxation steps were carried out for 125,000 steps using a 1-fs time step whereas the last three relaxation steps were carried out for 250,000 steps using a 2-fs time step. Temperature and pressure were controlled using the Berendsen thermostat and barostat (Berendsen et al., 1984), using semi-isotropic pressure coupling, a compressibility of 4.5 × 10^−5^ bar^−1^, and a coupling time constant of 5 ps. The production run was carried out for 1 μs (50,000,000 steps) using a 2-fs time step at 310 K and 1 bar pressure using the Nose-Hoover thermostat (Hoover, 1985; Nose, 1984) and the Parrinello-Rahman barostat (Parrinello and Rahman, 1981). Throughout the simulations, the electrostatic contributions were computed using the Particle Mesh Ewald algorithm (Darden et al., 1993) at a 1.2-nm cut-off while the van der Waals contributions were switched off between 1.0 and 1.2 nm. All heavy atom-hydrogen bonds were constrained using LINCS (Hess et al., 1997). The simulations were performed using Gromacs version 2021.5 (Abraham et al., 2015). To evaluate model stability, five repeats of 1 μs were carried out in both systems and the root-mean squared deviation from the initial energy-minimized structure was computed using the backbone atoms and excluding large loops.

### Building a coarse-grained model of yeast SPC

The CG model of each SPC variant (SPC with and without Spc2) was generated using the latest Martinize release (Kroon et al., 2023), employing sidechain fixes. Each protein monomer was constructed as a fully connected elastic network whose bonds had a force constant of 1,300 kJ mol^−1^ nm^−2^ within a distance cut-off of 0.8 nm. To stabilize the complex, intermolecular harmonic bonds were set using a force constant of 700 kJ mol^−1^ nm^−2^. The intermolecular bonds were set between PHE 44 in chain D and TRP88 in chain B, LEU64 in chain D and PHE 8 in chain B, LEU86 in chain A and THR112 in chain B, PRO135 in chain A and TRP161 in chain B, TYR10 in chain C and PRO 15 in chain B, and ALA5 in chain C and PHE 17 in chain A.

### Model validation

The coarse-grained RMSFs are obtained as the per-backbone bead average RMSF across the first 250 ns of five 20 μs repeats, with error bars showing the standard deviation, and atomistic RMSFs are obtained from C_α_ carbons during a 1 μs simulation. The RMSD was computed for the whole protein backbone while excluding large loops in VMD (Humphrey et al., 1996) starting from an energy minimized structure. To facilitate the task, the protein residues were renumbered and the selections used were as follows: “protein and not resid 262–271 and not resid 493–510 and not resid 242–261 and not resid 617–623” for the tetrameric variant and “protein and not resid 238–261 and not resid 317–330” for the trimer.

### Setup of coarse-grained MD simulations

Coarse-grained simulations were carried out using the Martini 3 Force Field (Souza et al., 2021). The SPC complex was embedded either into a POPC bilayer or into a symmetric endoplasmic reticulum membrane model using INSANE (Wassenaar et al., 2015) in a 15 × 15 × 15 cubic box, solvated in water, and 0.15 M NaCl. The ER membrane composition used is that proposed by Monje-Galvan and Klauda in 2015, DYPC:YOPC:POPI:PYPI:DYPE:YOPE:ERGO:YOPA:YOPS:POPS with ratios 42:28:21:14:10:10:7:6:6:6 (Monje-Galvan and Klauda, 2015). The principal axis of each SPC variant and the normal of the bilayer was then aligned to the z-axis.

All simulation boxes were first energy-minimized for 6,000 steps using the steepest descent and employing position restraint to the backbone beads. After minimization, two steps of equilibration were pursued using time steps of 5 and 10 fs, respectively. Both relaxation steps employed position restraints and ran for 50,000 steps at 310 K and 1 bar pressure. The temperature was kept constant using the Berendsen thermostat and the pressure coupling was handled by the Berendsen barostat (Berendsen et al., 1984), employing semi-isotropic pressure coupling and compressibility of 3 × 10^−4^ bar^−1^, which was applied independently to protein, membrane, and solvent. For production, five repeats of 20 µs each were carried out with a 20 fs time step, at 310 K and 1 bar pressure, using the velocity rescale thermostat (Bussi et al., 2007) and the Parrinello–Rahman barostat (Parrinello and Rahman, 1981) in Gromacs version 2021.5 (Abraham et al., 2015). To test whether model flexibility played a role in the thinning of the membrane within the TM-window of SPC, we built a more flexible model where the intermolecular contacts force constant was 70 mol^−1^ nm^−2^ smaller (fully flexible model) and a model fully connected with elastic bonds (rigid model). For the double mutant (Y79A,S83A) simulations, we used the intermediate flexibility model (with intermolecular contacts force constant of 700 mol^−1^ nm^−2^), and four repeats of 4-µs each were carried out.

### Membrane thickness

The membrane thickness in the TM window was calculated as the difference in the number density of PO4 beads in the TM versus the bulk obtained by fitting a triple Gaussian function to the SPC-containing simulations and a double Gaussian function to the bulk reference membrane, both in the POPC and ER membrane model systems. The TM window PO4s were taken as those 6 Å around the protein. A reference membrane was simulated for 1 µs for the bulk density value. For the all-atom simulations, the membrane thickness was computed by taking the phosphate atoms around 6 Å from the Cα atoms of the protein.

### Water density profiles and maps

The water density profiles were computed for each repeat using the gmx density tool. The water maps shown were obtained by computing the water densities using VolMap in VMD (Humphrey et al., 1996).

### Cross-angle

The cross-angle between the large helix of Spc3 and the helix defined between residues 24 and 43 in Spc1 was calculated using a Python script based on MDAnalysis (Gowers et al., 2019; Michaud-Agrawal et al., 2011) and numpy (Harris et al., 2020). The cross angle was calculated as the angle between the vector described from the three first and three last residues in the Spc3 helix defined between residues 20 and 4 and the vector described from the three first and three last residues in the Spc1 helix defined between residues 24 and 43.

### Online supplemental material

Fig. S1 presents sequences and data related to Spc2, its homologs, and mutants. Fig. S2 shows the predicted signal sequence cleavage sites of the proteins used in the study. Fig. S3 provides model validation for the MD simulations. Fig. S4 includes MD simulation data on water density profiles and maps. Fig. S5 illustrates structural rearrangements of the SPC upon removal of Spc2. Table S1 lists the signal sequences used in Fig. 1 and Fig. 3 E. Data S1 contains mass spectrometry data on the abundance ratio between *spc2*Δ and WT yeast strains. Data S2 includes mass spectrometry data comparing the abundance ratios in *spc2*Δ strains carrying *spc2-ΔCD*(*58*), spc2-TM2* or *SPC2*.

## Supplementary Material

Table S1 shows signal sequences used in Fig. 1 and Fig. 3 E.

Data S1 shows mass spectrometry data on the abundance ratio between *spc2*Δ and WT yeast strains.

Data S2 shows mass spectrometry data comparing the abundance ratios in *spc2*Δ strains carrying *spc2-ΔCD*(*58*), spc2-TM2* or *SPC2*.

SourceData F1 is the source file for Fig. 1.

SourceData F2 is the source file for Fig. 2.

SourceData F3 is the source file for Fig. 3.

SourceData F4 is the source file for Fig. 4.

SourceData F6 is the source file for Fig. 6.

SourceData FS1 is the source file for Fig. S1.

## Data Availability

All data are included in the article and supplemental materials or are available from the corresponding author upon reasonable request.
