# Supplementary material for: Spc2 modulates substrate- and cleavage site-selection in the yeast signal peptidase complex
Source: J Cell Biol. 2024 Nov 20;223(12):e202211035. doi: 10.1083/jcb.202211035 (PMC11579918; doi:10.1083/jcb.202211035)
Supplement: Table S1 — shows signal sequences used in Fig. 1 and Fig. 3 E. [file jcb_202211035_tables1.docx]

**Table S1. Signal sequences used in Fig.1 and 3E**

| **Name** | **N-terminal signal sequences** | **N-length (aa)** |
| --- | --- | --- |
| N0CPYt(*h*) | MKLLLTLLLCLLLLSTTLAKAISL | 0 |
| N9CPYt(*h*) | MKKKHLLDKLLLTLLLCLLLLSTTLAKAISL | 9 |
| N12CPYt(*h*) | MFDTKKKHLLDKLLLTLLLCLLLLSTTLAKAISL | 12 |
| N16CPYt(*h*) | MPDELFDTKKKHLLDKLLLTLLLCLLLLSTTLAKAISL | 16 |
| N20CPYt(*h*) | MVERIPDELFDTKKKHLLDKLLLTLLLCLLLLSTTLAKAISL | 20 |
| N24CPYt(*h*) | MGEEEVERIPDELFDTKKKHLLDKLLLTLLLCLLLLSTTLAKAISL | 24 |
| N26CPYt(*h*) | MEGGEEEVERIPDELFDTKKKHLLDKLLLTLLLCLLLLSTTLAKAISL | 26 |
| SP_suc_-Lep | MLLQAFLFLLAGFAAKISASMGSM | 0 |
| ppαF | MRFPSIFTAVLFAASSALAAPVNTTTE | 0 |
| Ecm38 | MLLCNRKVPKTLNTCFILHIFTLLTLGVLVSGMPSKMV | 10 |
| Kar2 | MFFNRLSAGKLLVPLSVVLYALFVVILPLQNSFH | 10 |
| Pho8(P54A) | MMTHTLPSEQTRLVPGSDSSSRPKKRRISKRSKIIVSTVVCIGLLLVLVQLAF**A**SSFA | 32 |

N-length indicates the number of amino acids (aa) preceding the hydrophobic core of the signal sequence (underlined). In N#CPYt(*h*) variants, # indicates *n*-region length (N-length), t denotes a C-terminal truncation after residue 323 of CPY for better separation of protein products with cleaved and uncleaved signal sequence on SDS-PAGE, and *h* denotes hydrophobic variant of CPY. Pho8(P54A) sequence is shown with A in bold.
