# Supplementary figures and images for: Spc2 modulates substrate- and cleavage site-selection in the yeast signal peptidase complex
Source: J Cell Biol. 2024 Nov 20;223(12):e202211035. doi: 10.1083/jcb.202211035 (PMC11579918; doi:10.1083/jcb.202211035)

SourceDataF1B

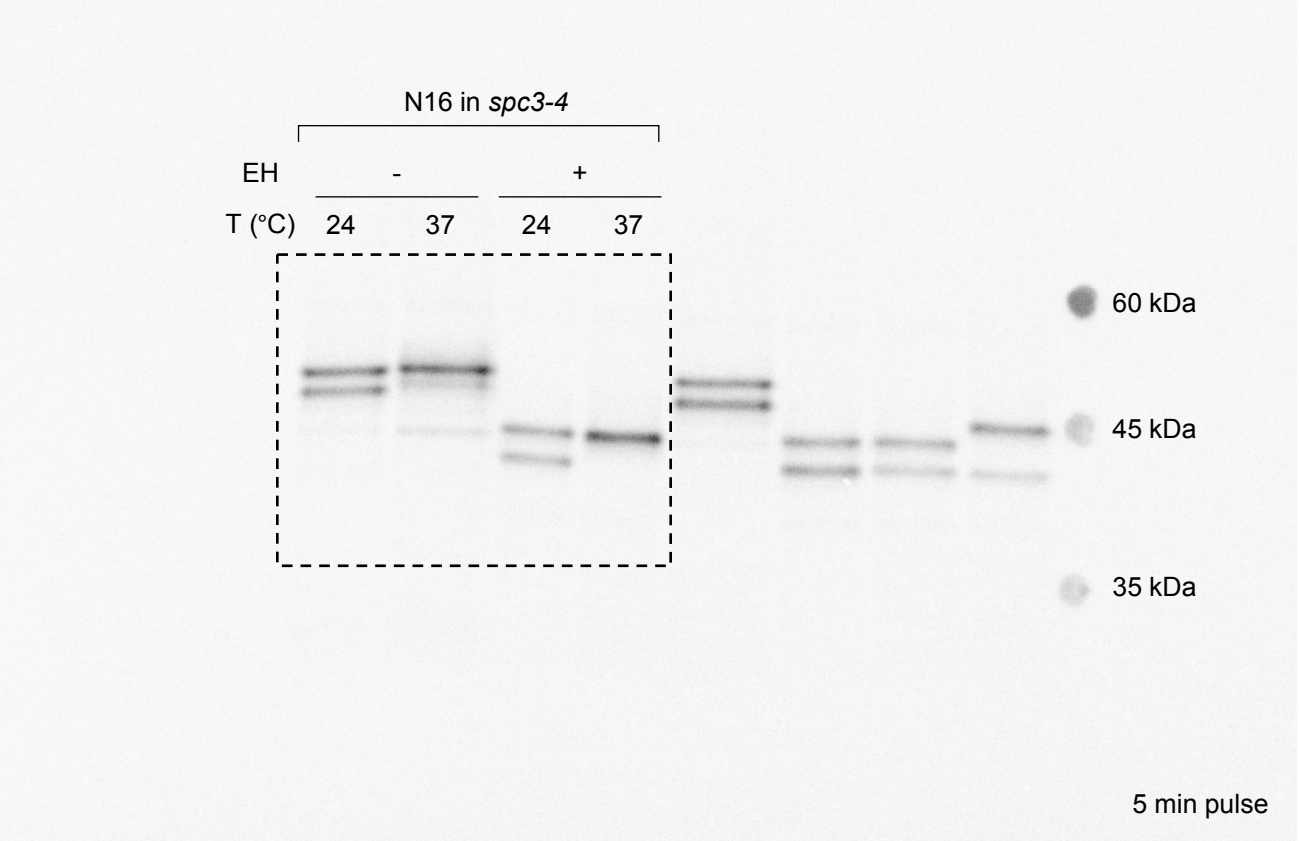

# Source Data Figure 1E

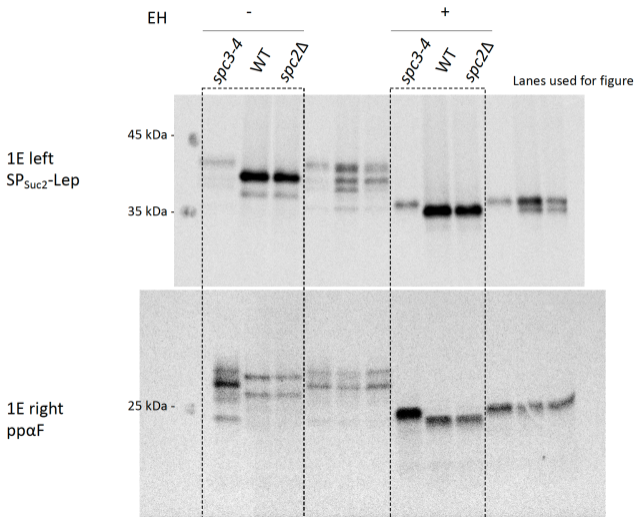

SourceDataF1F

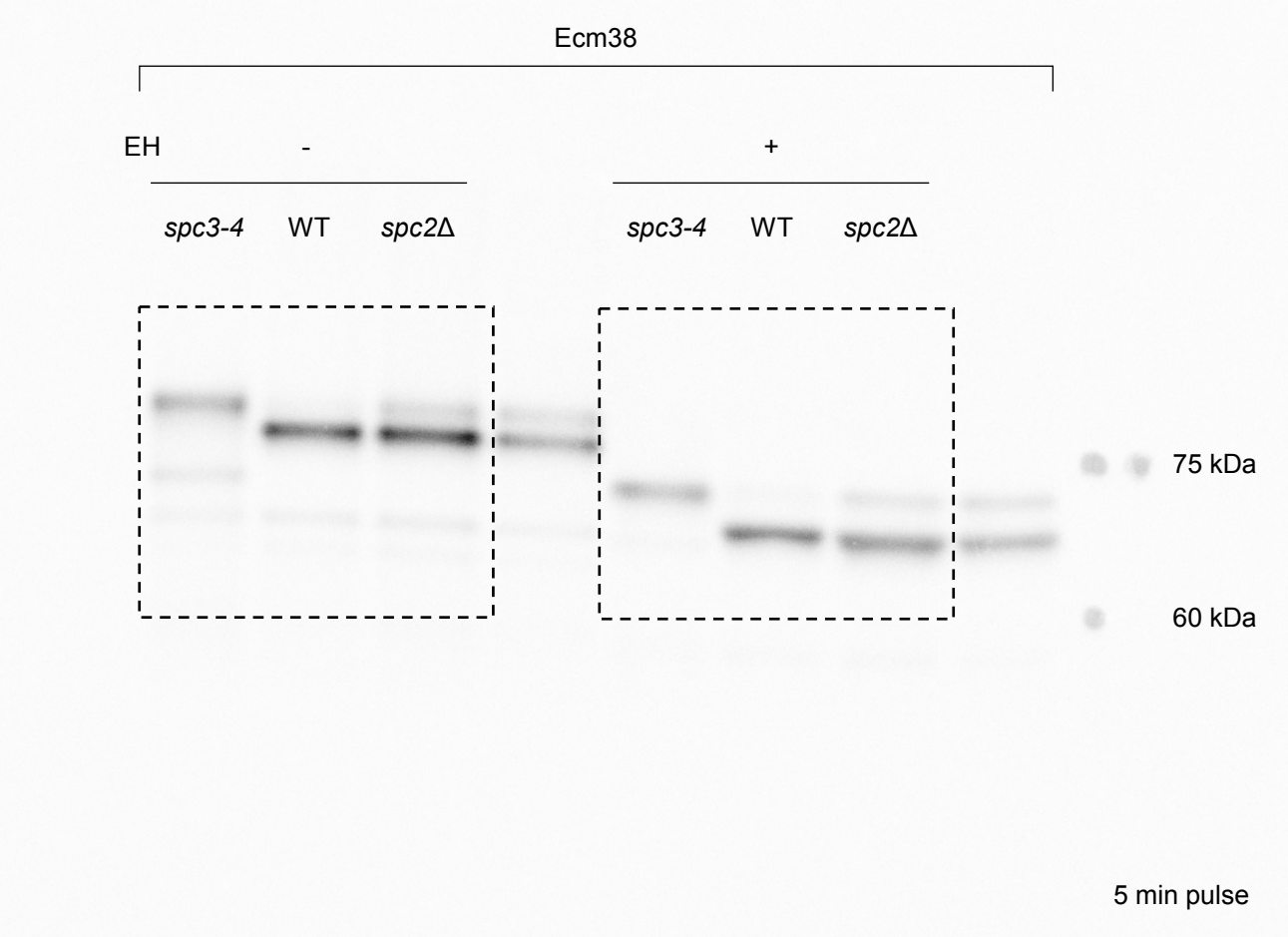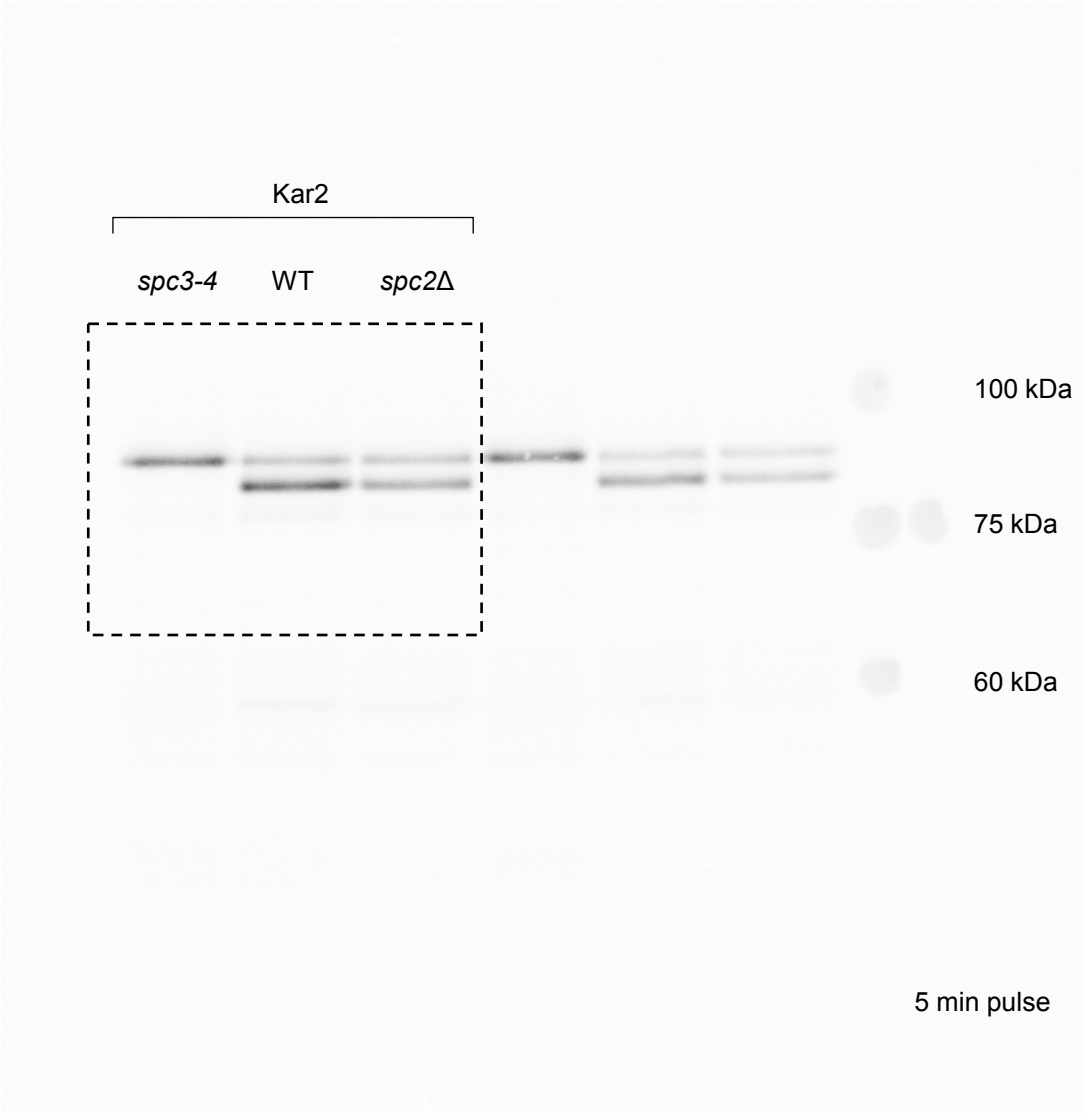

Supplement: SourceData F1 — is the source file for Fig. 1. [file jcb_202211035_sourcedataf1.pdf]

## Source Data Fig 2D

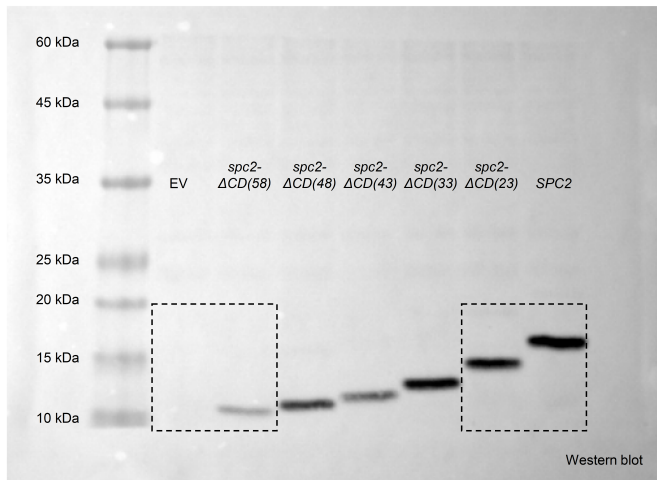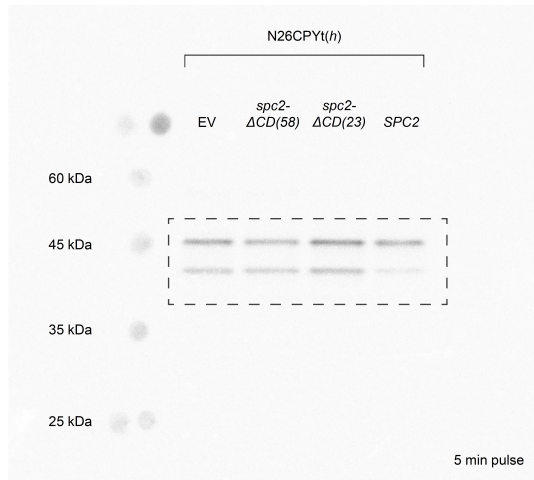

*spc2Δ SPC3HA*

Source Data Fig 2E

EV

SPC2

*spc2-ΔCD(58)*

*spc2-TM2\**

35 kDa

20 kDa

45 kDa

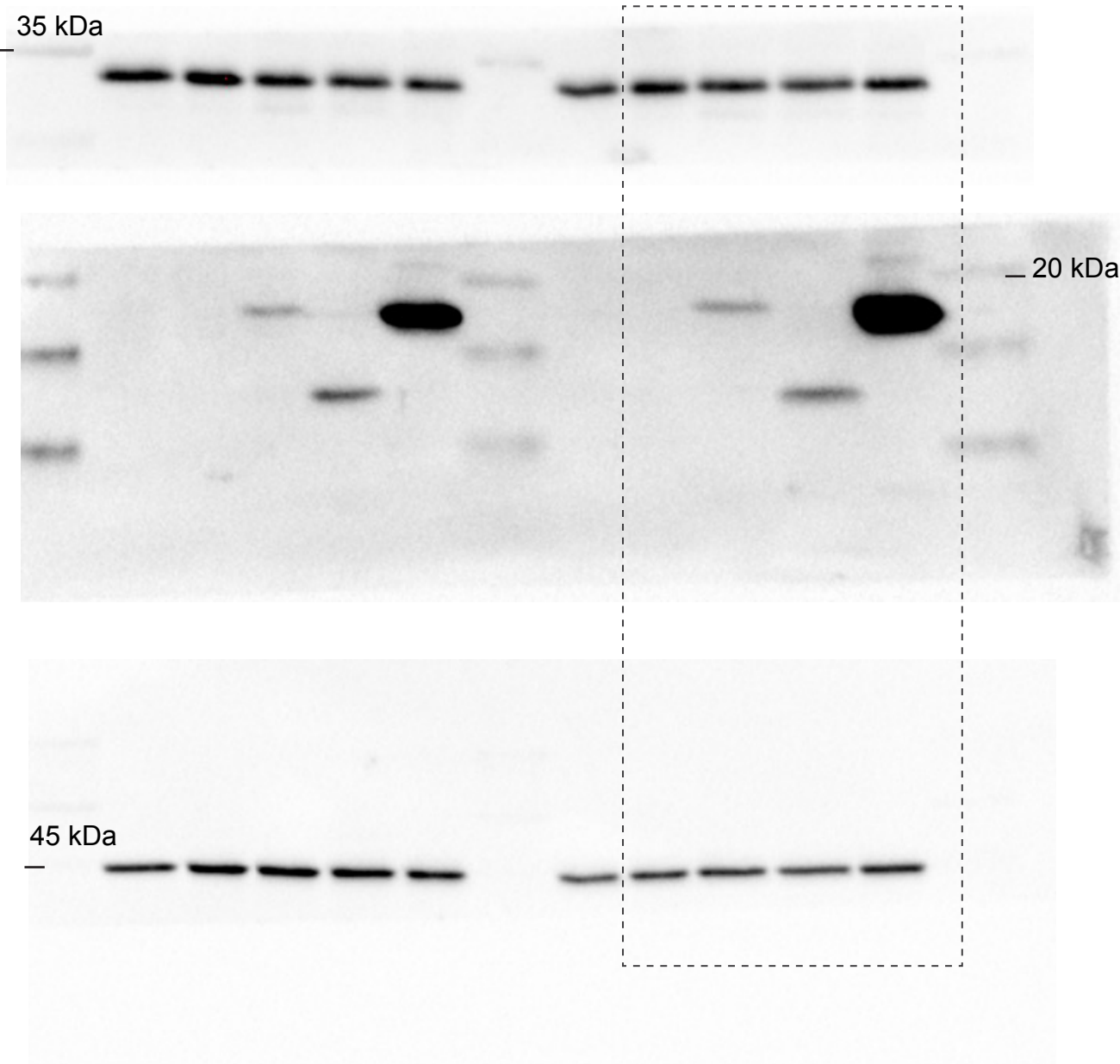

Supplement: SourceData F2 — is the source file for Fig. 2. [file jcb_202211035_sourcedataf2.pdf]

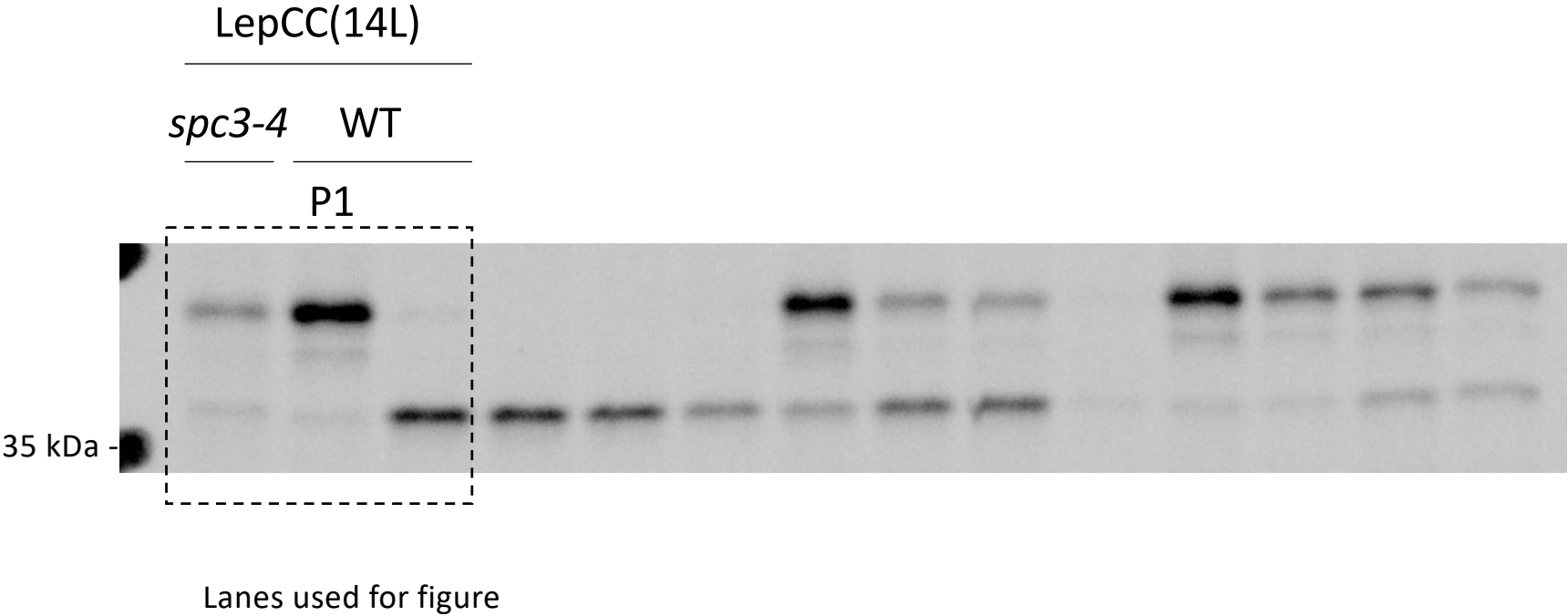

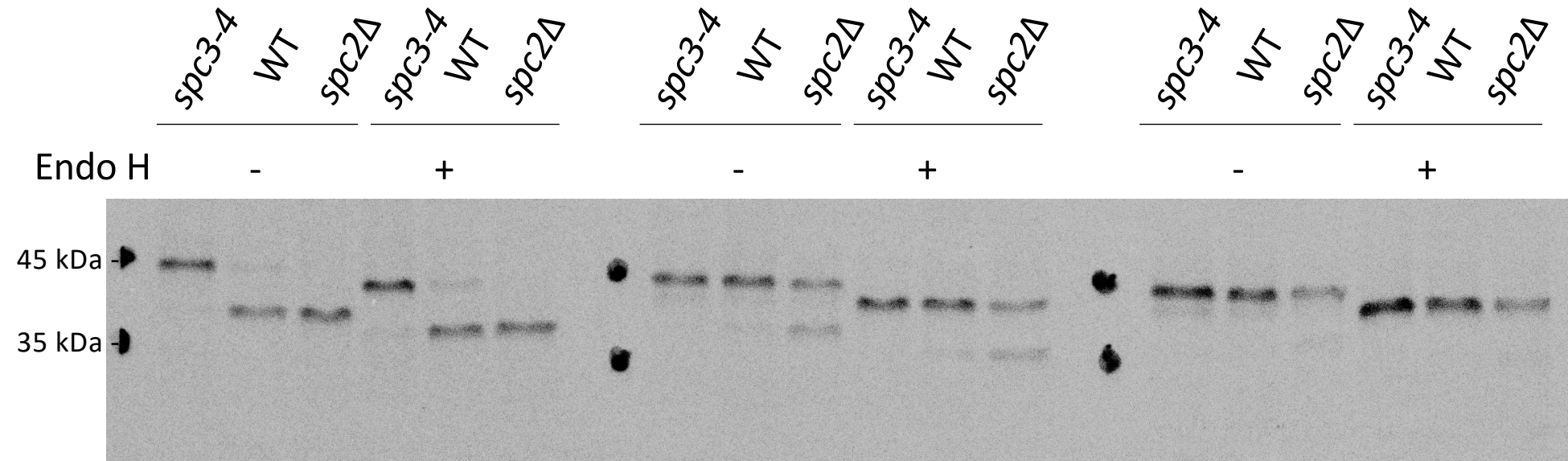

SourceDataF3E

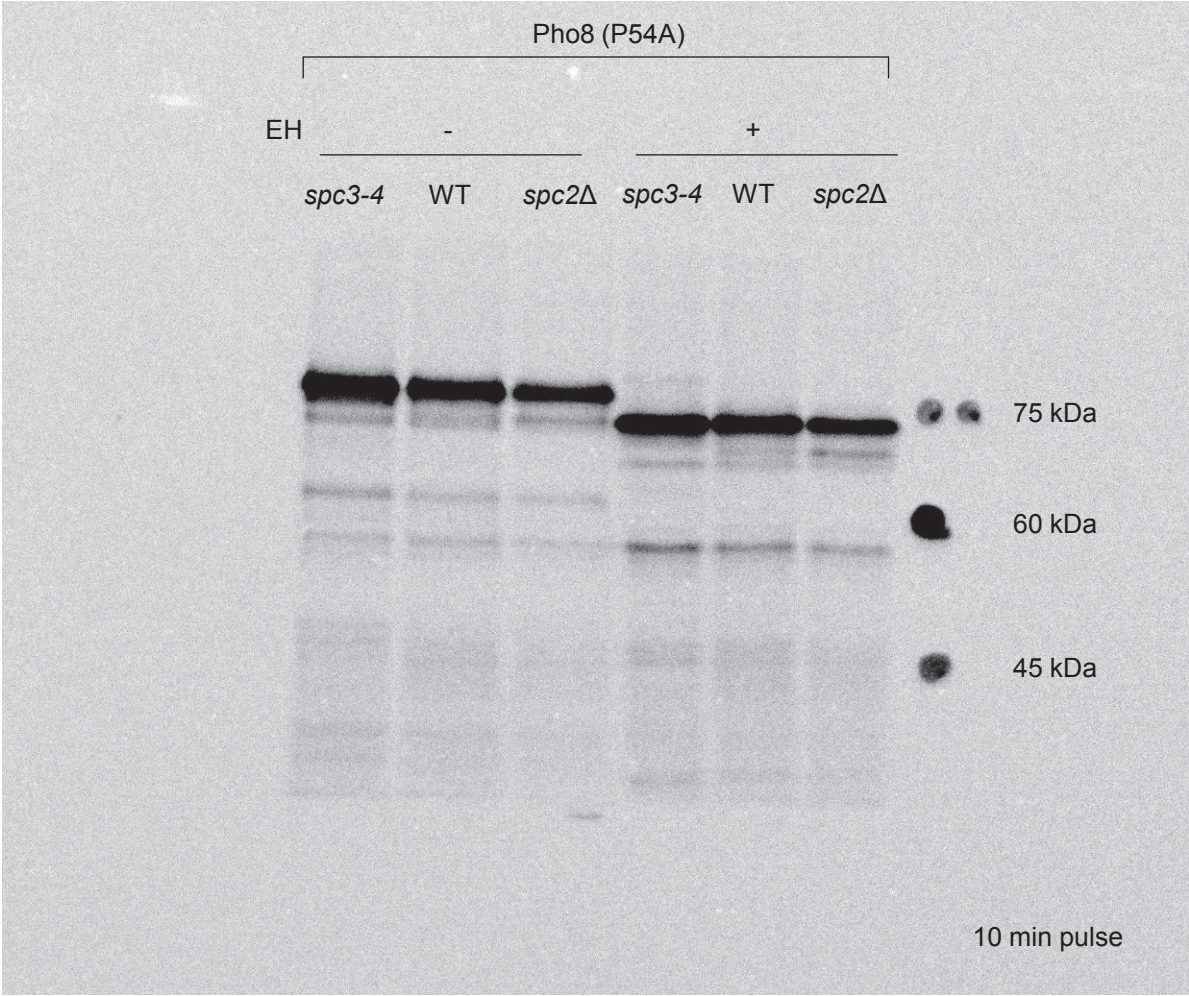

Supplement: SourceData F3 — is the source file for Fig. 3. [file jcb_202211035_sourcedataf3.pdf]

SourceDataF4B

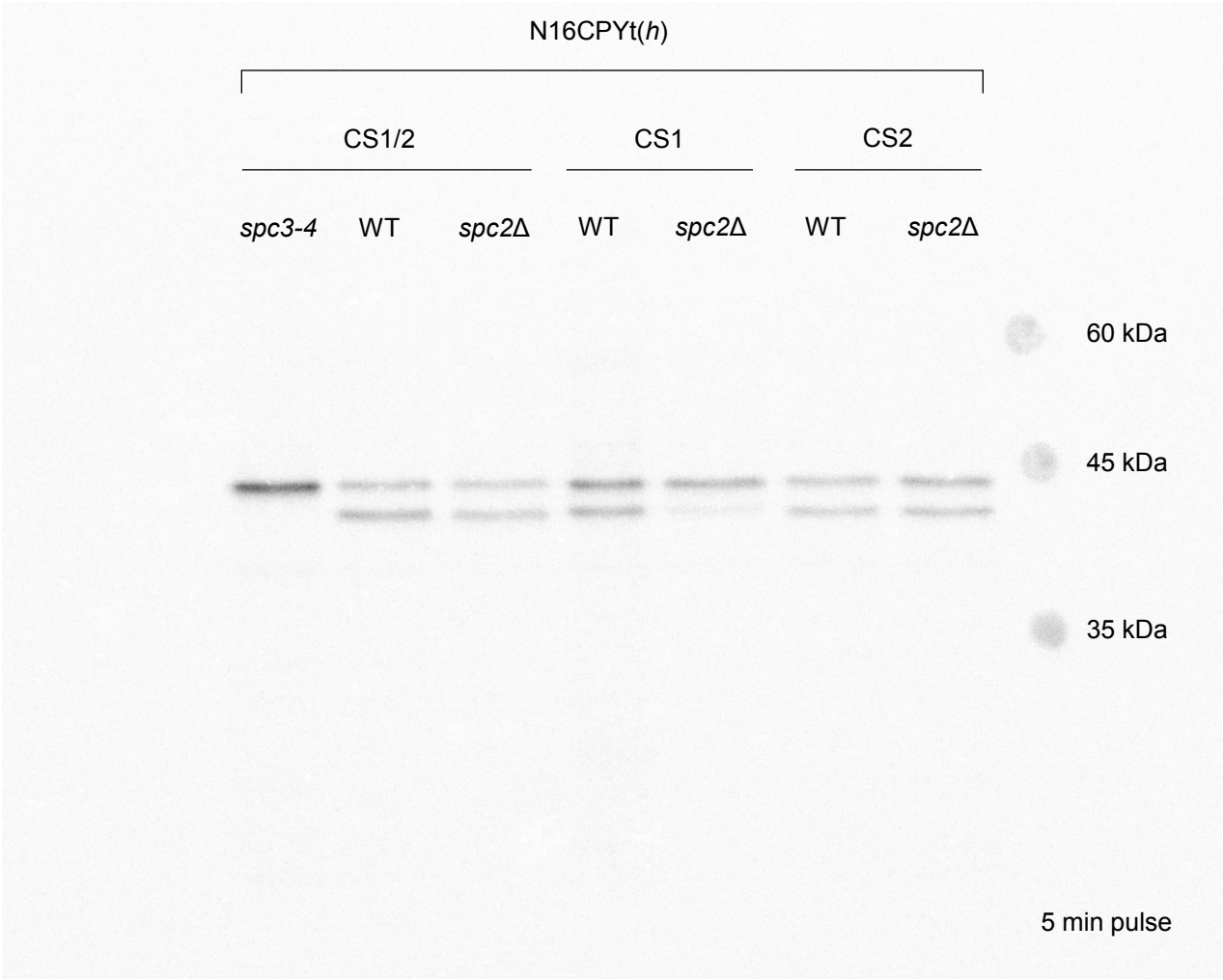

# Source Data Fig 4C

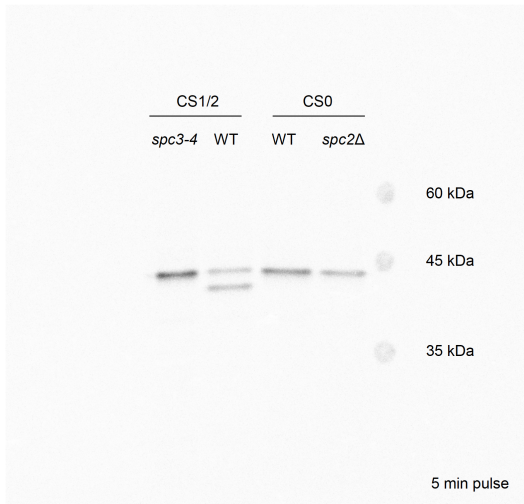

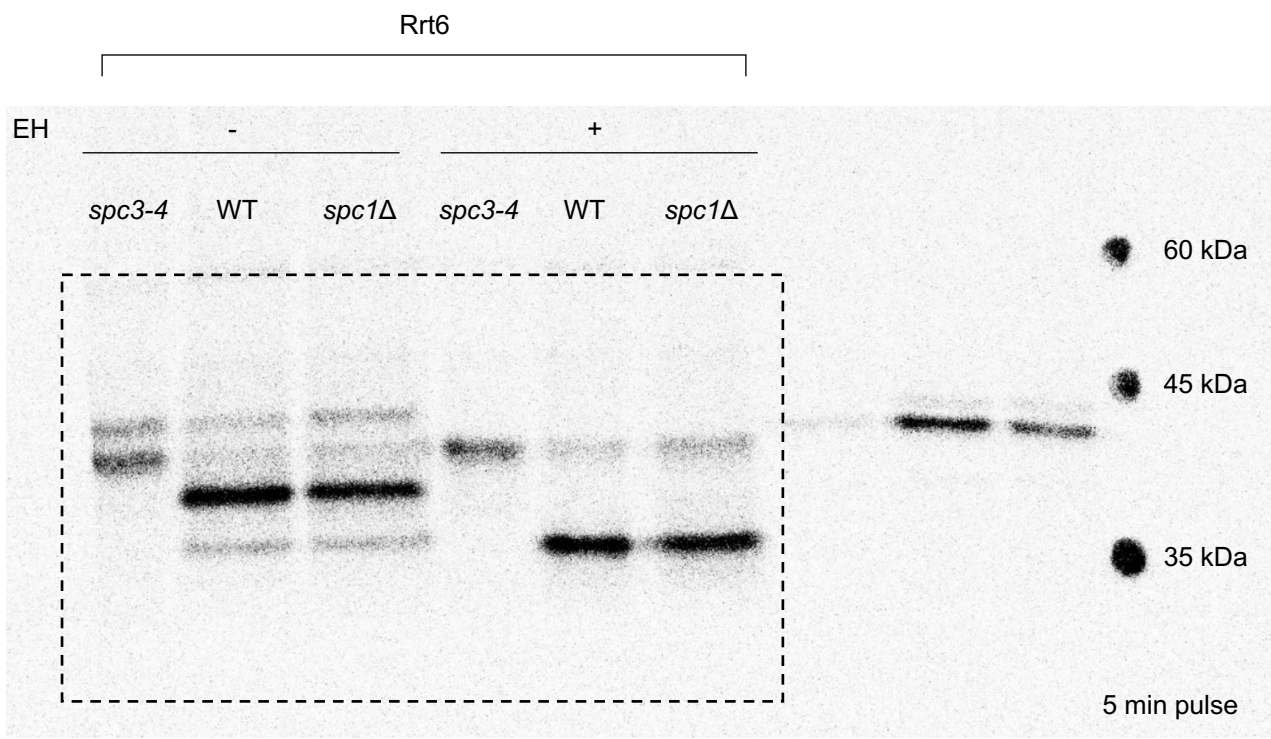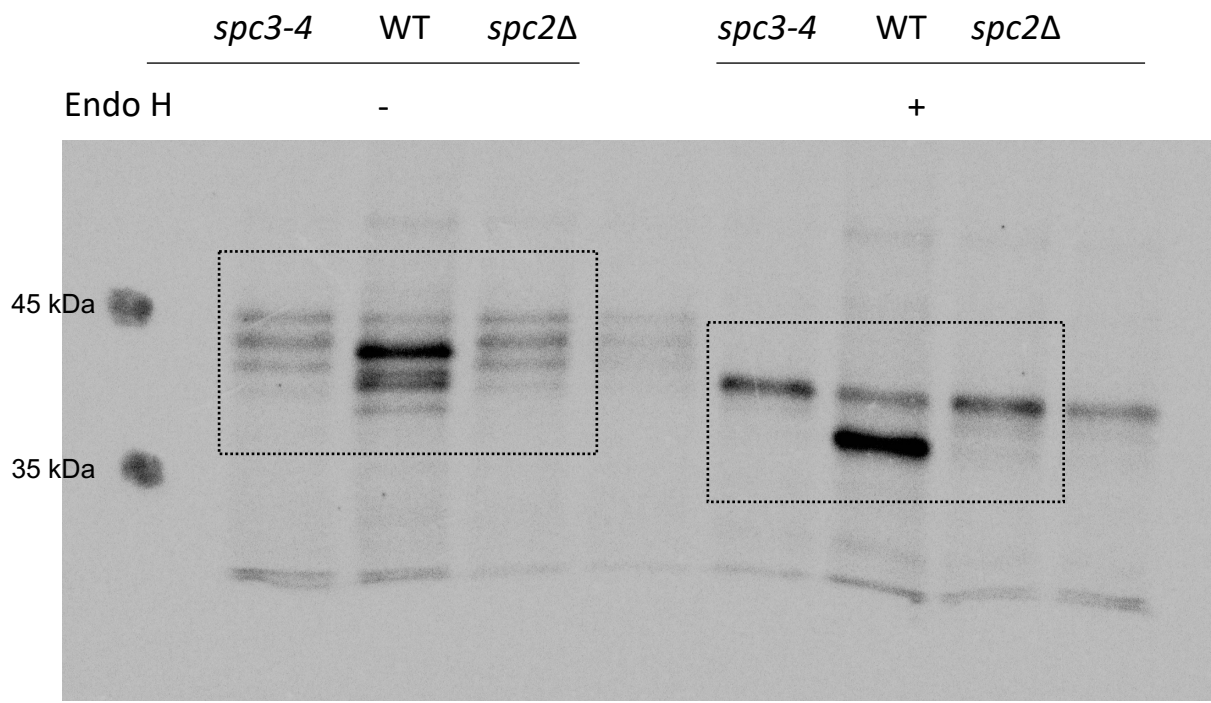

Source Data Fig 4G

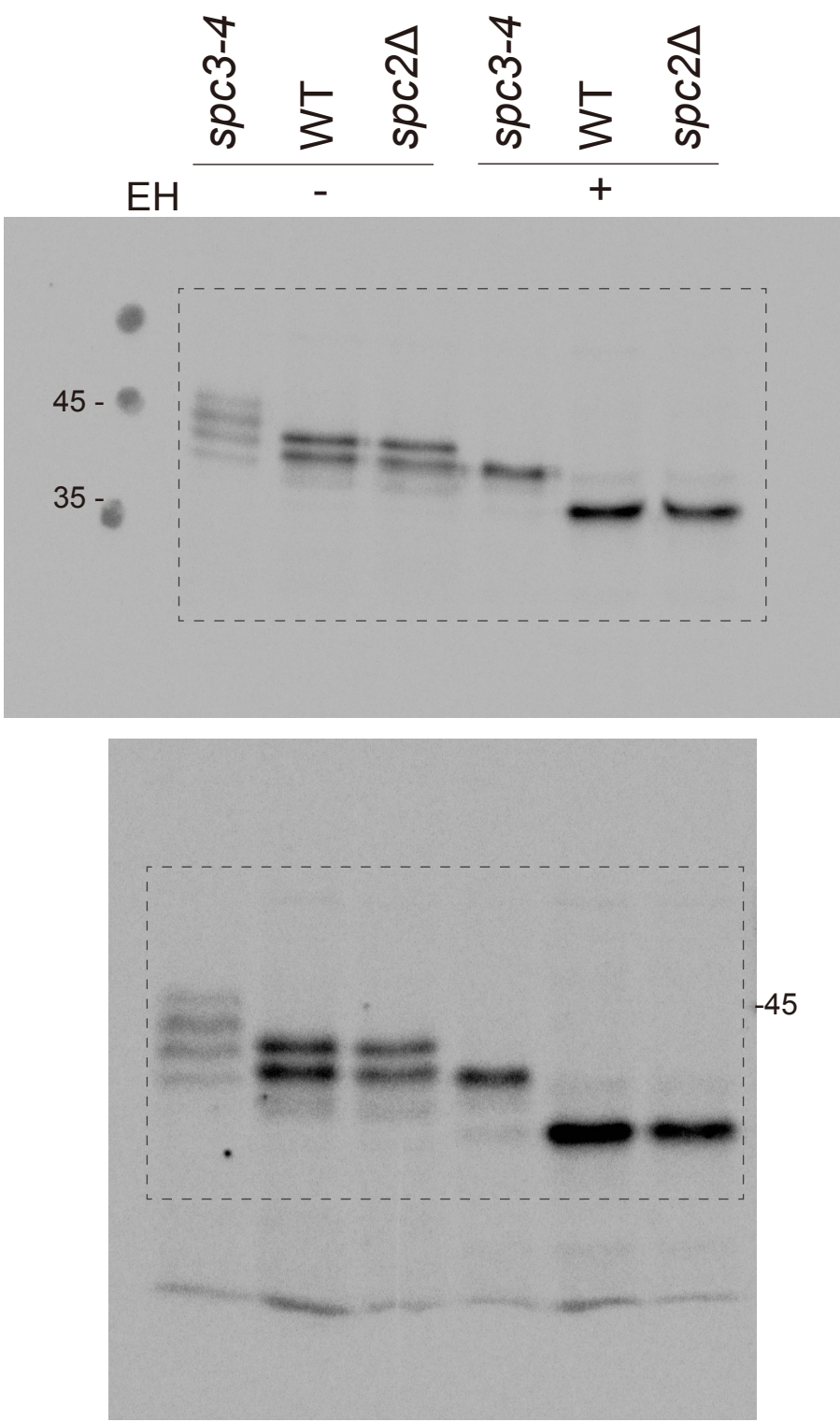

Supplement: SourceData F4 — is the source file for Fig. 4. [file jcb_202211035_sourcedataf4.pdf]

## Source Data Fig 6B

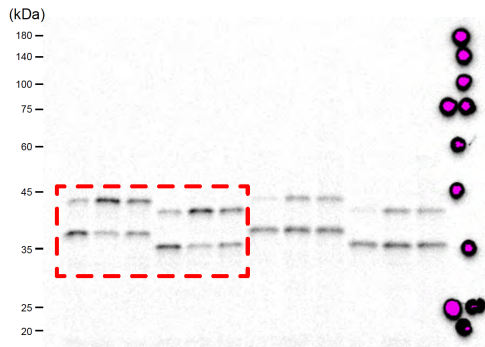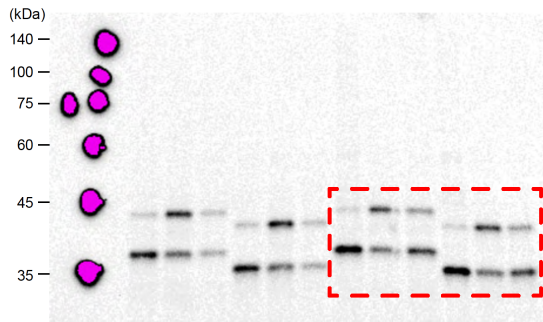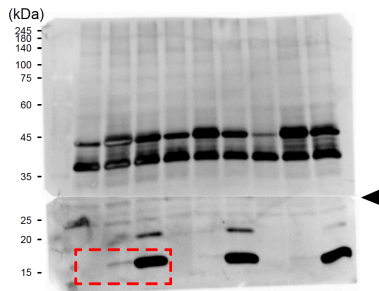

Supplement: SourceData F6 — is the source file for Fig. 6. [file jcb_202211035_sourcedataf6.pdf]

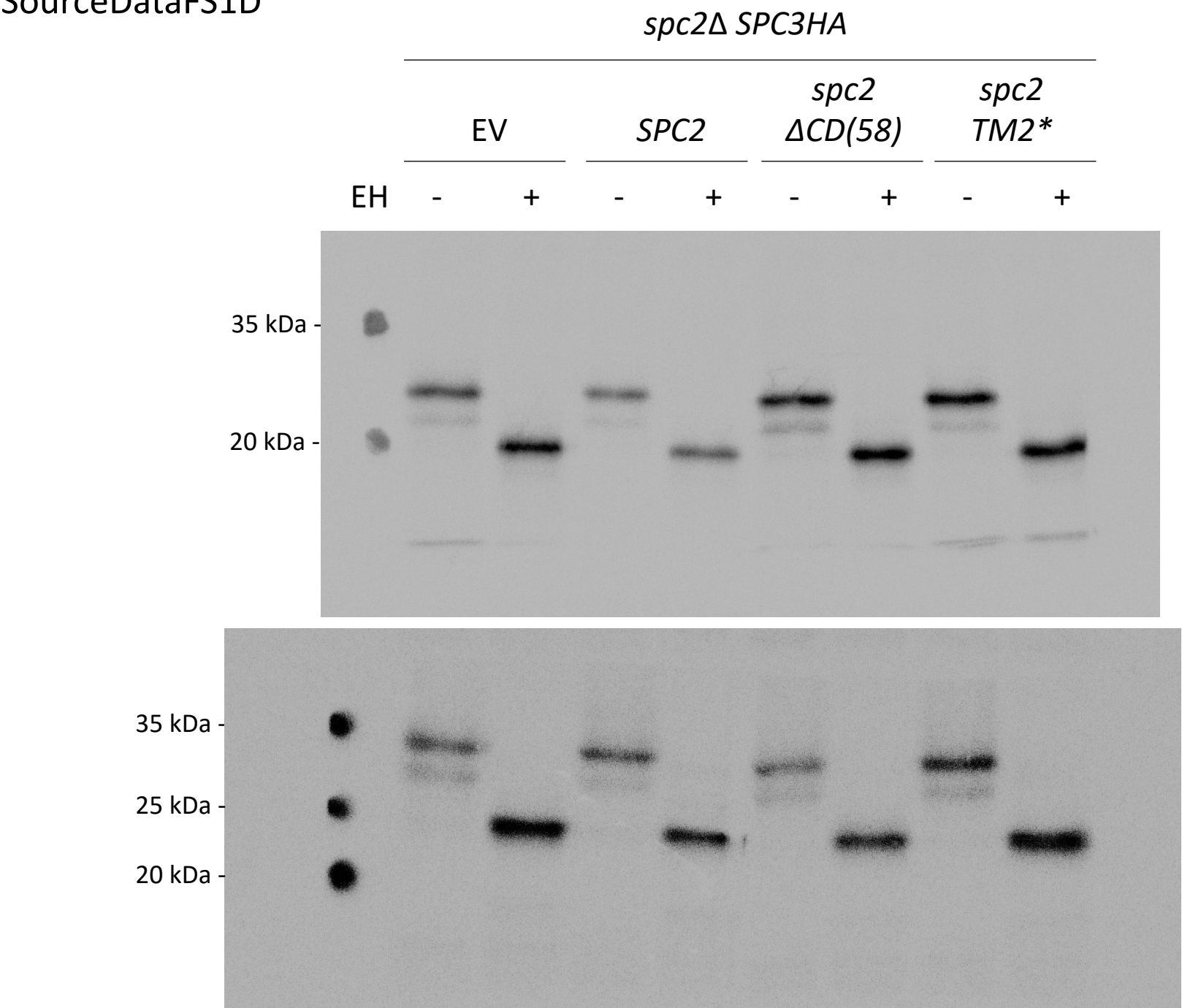

Supplement: SourceData FS1 — is the source file for Fig. S1. [file jcb_202211035_sourcedatafs1.pdf]
